# A novel cell nuclei segmentation method for 3D *C. elegans* embryonic time-lapse images

**DOI:** 10.1186/1471-2105-14-328

**Published:** 2013-11-19

**Authors:** Long Chen, Leanne Lai Hang Chan, Zhongying Zhao, Hong Yan

**Affiliations:** 1 Department of Electronic Engineering, City University of Hong Kong, Kowloon, Hong Kong; 2 Department of Biology, Hong Kong Baptist University, Kowloon, Hong Kong

## Abstract

**Background:**

Recently a series of algorithms have been developed, providing automatic tools for tracing *C. elegans* embryonic cell lineage. In these algorithms, 3D images collected from a confocal laser scanning microscope were processed, the output of which is cell lineage with cell division history and cell positions with time. However, current image segmentation algorithms suffer from high error rate especially after 350-cell stage because of low signal-noise ratio as well as low resolution along the Z axis (0.5-1 microns). As a result, correction of the errors becomes a huge burden. These errors are mainly produced in the segmentation of nuclei. Thus development of a more accurate image segmentation algorithm will alleviate the hurdle for automated analysis of cell lineage.

**Results:**

This paper presents a new type of nuclei segmentation method embracing an bi-directional prediction procedure, which can greatly reduce the number of false negative errors, the most common errors in the previous segmentation. In this method, we first use a 2D region growing technique together with the level-set method to generate accurate 2D slices. Then a modified gradient method instead of the existing 3D local maximum method is adopted to detect all the 2D slices located in the nuclei center, each of which corresponds to one nucleus. Finally, the bi-directional pred- iction method based on the images before and after the current time point is introduced into the system to predict the nuclei in low quality parts of the images. The result of our method shows a notable improvement in the accuracy rate. For each nucleus, its precise location, volume and gene expression value (gray value) is also obtained, all of which will be useful in further downstream analyses.

**Conclusions:**

The result of this research demonstrates the advantages of the bi-directional prediction method in the nuclei segmentation over that of StarryNite/MatLab StarryNite. Several other modifications adopted in our nuclei segmentation system are also discussed.

## Background

The development of the live-cell imaging microscopy and fluorescent tagging provides us unprecedented opportunity to observe gene expression, nuclei movement and nuclei division processes during embryogenesis at the single cell level [1-3]. Customized algorithms were developed using high temporal resolution of image stacks to automatically trace cell divisions during animal development.

One system named StarryNite has been developed for automated cell lineage tracing and gene expression profiling during *C.elegans* embryogenesis [4,5]. A lineage tree can be generated with StarryNite automatically based on the 3D time-lapse image of a developing *C. elegans* embryo. To obtain cell nuclei with high signal/noise ratio, the *C. elegans* embryos are first tagged with a fluorescent protein. Then the authors took images every 60 or 90 seconds with a confocal microscope during *C. elegans* embryogenesis. At every time point there are 41 image planes and the resolution in the Z-axis is 0.71 microns. A complete 3D time-lapse image series contains 180–240 time points when the cell number of the embryo is over 500. So there are over 6000 images in a single image series, which record approximately 600 cell divisions and 50000 nuclei at different time points. With these images as input to StarryNite system, information on reporter expression, nucleus division and movement can be efficiently extracted from a huge mass of image data. [Additional file 1: Table S1].

However, due to the low resolution in the Z-axis and high noise in the 3D time-lapse image of *C. elegans*, StarryNite/MatLab StarryNite output suffers from high error rate especially during late embryogenesis. It takes around 2–5 human hours for manually annotating StarryNite’s output up to 350 cell stage, but for most cells, at least one division process cannot be correctly traced in this kind of lineage tree [6-8].

StarryNite/MatLab StarryNite can be generally divided into two parts, namely, the nuclei segmentation part and the tracing part. The nuclei segmentation part will analyze the 3D time-lapse images of *C. elegans* and detect the nuclei positions, size and intensity at every time point. Then the tracing part will build the lineage tree according to the nuclei information generated by the nuclei segmentation part. The main reason for StarryNite/MatLab StarryNite not being able to reach the 550 cell stage, with an acceptable error rate, is that too many cells could not be detected by the nuclei segmentation algorithm that works relatively well at or before the 350 cell stage. In fact, the detection accuracy of the nuclei segmentation part in StarryNite can only work for up to 350 cells at any one single time point (MatLab StarryNite, 500 cells). Therefore at the end of embryogenesis (cell number at 550), more than 200 cells cannot be detected correctly. Therefore, for every 3D image data set, even if the tracing part is error free, the number of errors would still be more than 7000, which means that one to two weeks of manual editing time are needed for generation of a 550-celled lineage. Therefore, development of a novel or optimization of the existing nuclei segmentation algorithm is the key to speed up the automatic lineaging throughout *C. elegans* embryogenesis.

In order to improve the performance of StarryNite, several techniques have been developed and implemented into StarryNite. Santella et al. [9] presents a blob-slice nuclei segmentation model to improve the correct nuclei detection rate and this model can be also used to analyze 3D images of other species, such as zebrafish and Drosopila [9]. Aydin builds a new tracing system with a SVM classifier, which could greatly reduce the number of false positive errors that can be detrimental during the editing of the lineage tree [10]. Richards et al. [11] increase the 3D time-lapse image resolution in the Z-axis with a resonance-scanning confocal microscope [11]. In his work, the lineage tree generated by Matlab StarryNite with these new images could reach the 500 cell stage with manual editing. In all the above-mentioned methods, the 3D local maximum is a must for the nuclei segmentation, based on the principle that one particular 3D local maximum point in images corresponds to a certain nucleus. However, as the number of images increases, nuclei at the top of the embryo will be flattened and crowded. Consequently, one 3D local maximum point in images at this stage cannot always indicate one nucleus but two (or more) nuclei. Besides, in 3D local maximum, there are several other techniques available for 3D nuclei segmentation such as adaptive thresholds, [12,13] mode finding, [14,15] gradient flow tracking, [16] watershed [17,18] and level set methods [19]. These methods perform well when nuclei are widely spaced, but given conditions in which nuclei are crowded, as in the low resolution along the Z axis in the 3D image, all these methods show some disfigurement to various degrees.

Judging from these aspects, we speculate that under certain circumstances, all the above methods have difficulty in finding certain nuclei at some time points due to the low image quality. In view of this issue, we develop a technique called bi-directional prediction to reduce false negative errors and improve the precision and accuracy of nuclei segmentation. Due to the minimum position shift of a nucleus between two adjacent time points, if there is a nucleus that can hardly be detected based on the image of one single time point, we can predict it by studying the images before and after the current time point.

In this paper, a new nuclei segmentation method is attempted to substitute that for StarryNite, which enables a more accurate extraction of useful information from 3D time-lapse images. The results achieved by our new nuclei segmentation method demonstrate better performance in nuclei segmentation and reduced false negative errors. Currently, editing a lineage up to 350 or 450 cell stage requires roughly 0.5 or 8 hours respectively using our image series.

## Result

### Error rate analysis

The error statistics of the StarryNite/MatLab StarryNite results of two data sets (130108PHA4p1 and 130108NHR25p1) are studied in our work. As shown in Table 1, errors produced by StarryNite can be divided into two categories: (1) Nuclei segmentation errors, (2) Tracing errors.

**Table 1:** **Error analysis of StarryNite**[4]

**Dataset**	**Segmentation errors**				**Tracing errors**	**Total errors**	**Total cell number**
	**False positives**	**False negatives**	**False diameters**	**Dislocations**			
081505	111	297	132	150	237	927	5007
130108PHA4p1	233	713	290	240	308	1784	13455
130108NHR25p1	204	760	253	168	284	1669	15595
Total	548	1770	675	558	829	4380	34057

081505: 1–99 time points (1 minute per time point),

130108PHA4p1 and 130108NHR25p1: 1–135 time points (1.5 minutes per time point).

The main source of nuclei segmentation errors includes failure to identify nuclei (false negatives), misjudgments of non-existent nuclei (false positives), false diameter errors and dislocation errors. As illustrated in Table 1, false negative errors are the most common errors in the StarryNite/MatLab StarryNite output. What is more important is that nuclei segmentation errors will propagate to tracing errors. In fact, the majority of tracing errors in the StarryNite/MatLab StarryNite output which do not detect matching errors and false division identification errors, are caused by nuclei segmentation errors like dislocations or false diameters. According to the above analysis, it is the nuclei segmentation errors, especially the false negative errors, that become the biggest stumbling barrier in using the StarryNite/MatLab StarryNite system. For this reason we have built a new nuclei segmentation system which focuses on eliminating false negative errors.

There are three reasons why the multiplication of false negative errors occurs at the end of the embryonic development. First, the image resolution in the Z-axis is 0.71 microns, which means that one small nucleus (with nucleus diameter < 1.8 microns) can be clearly seen in only two images. Second, the increase in nuclei numbers makes the nuclei at the top of the embryos become flattened and crowded to such an extent that they are not always recovered by the iterative nuclei identification.^4^ This crowding becomes a significant source of errors after the 150-cell stage, when the nuclei first enter the very top of the embryo. Third, neighboring cells may have different levels of GFP expression. When two nuclei are close to each other, the local signal density of the weaker one may blend into the edge of the high signal from the brighter one and, thus, no longer obey the rule of local maximum. Accordingly, we designed our nuclei segmentation system for the purpose of avoiding false negative errors from the above three original sources of error formation.

### Algorithm design

**Figure 1:**
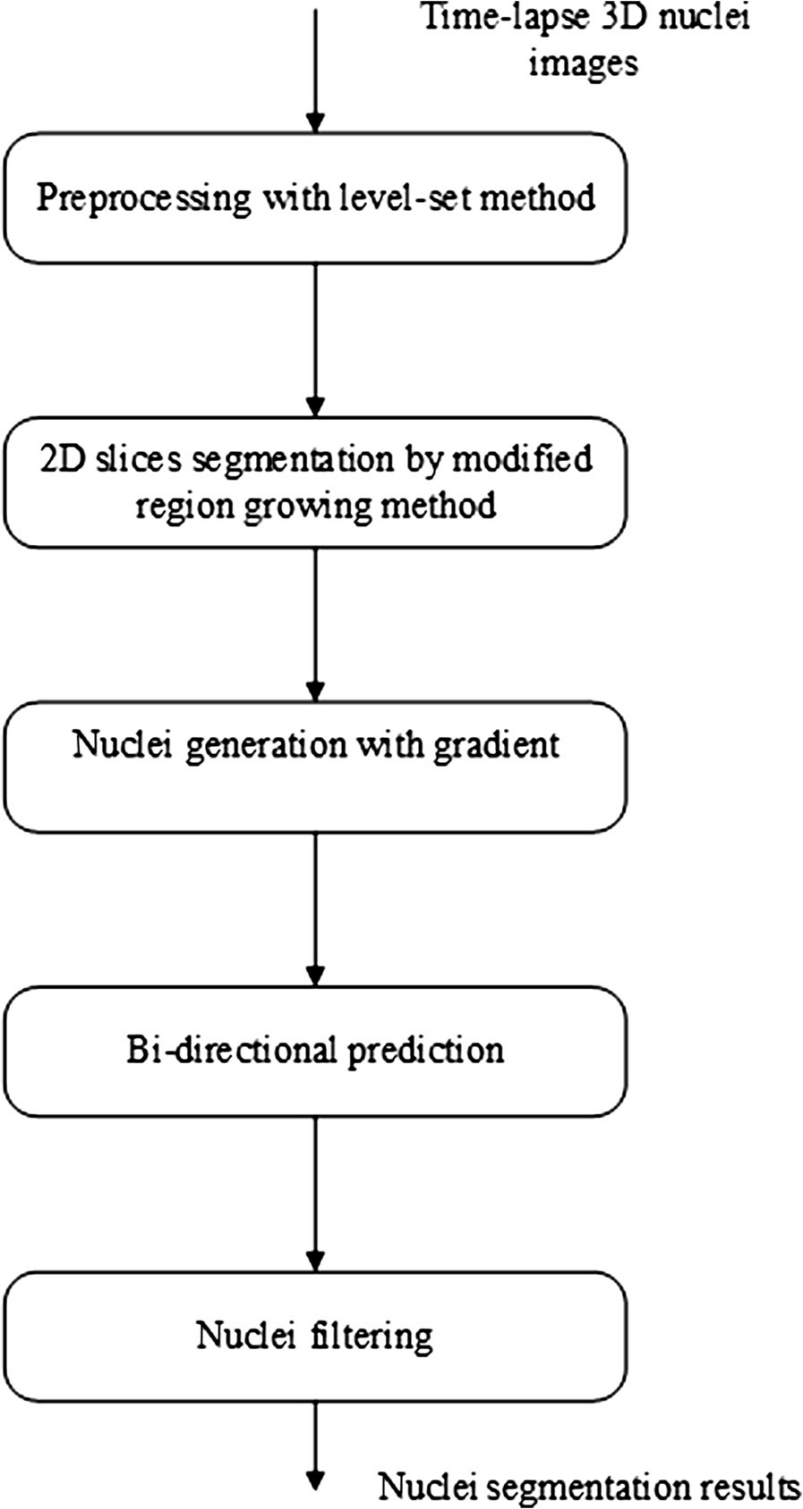
Flowchart of our nuclei segmentation system.

Figure 1 provides a flow of our method, which can be divided into five main steps.

#### Step1: Pre-processing with level-set method (LSM)

There are several limitations in the 3D time-lapse images of *C. elegans* embryo: First, the image resolution in the Z-axis is 0.71 microns, while the diameter of some nuclei at the 450 cell stage is around 1.8 microns, so each of these small nuclei can only be mapped in one or two images. These defects cause extraordinary difficulties in nuclei detection. Second, the GFP expression of some nuclei are quite low, so distinguishing the noises of the original images from a low GFP expression signal is a major issue. Third, the nuclei boundaries are not clear, because of the crowed nuclei at the 450 cell stage, thus a boundary enhancement method is needed for an accurate segmentation. In our work we overcome the above difficulties by pre-processing the images with the level-set method before segmentation.

**Figure 2:**
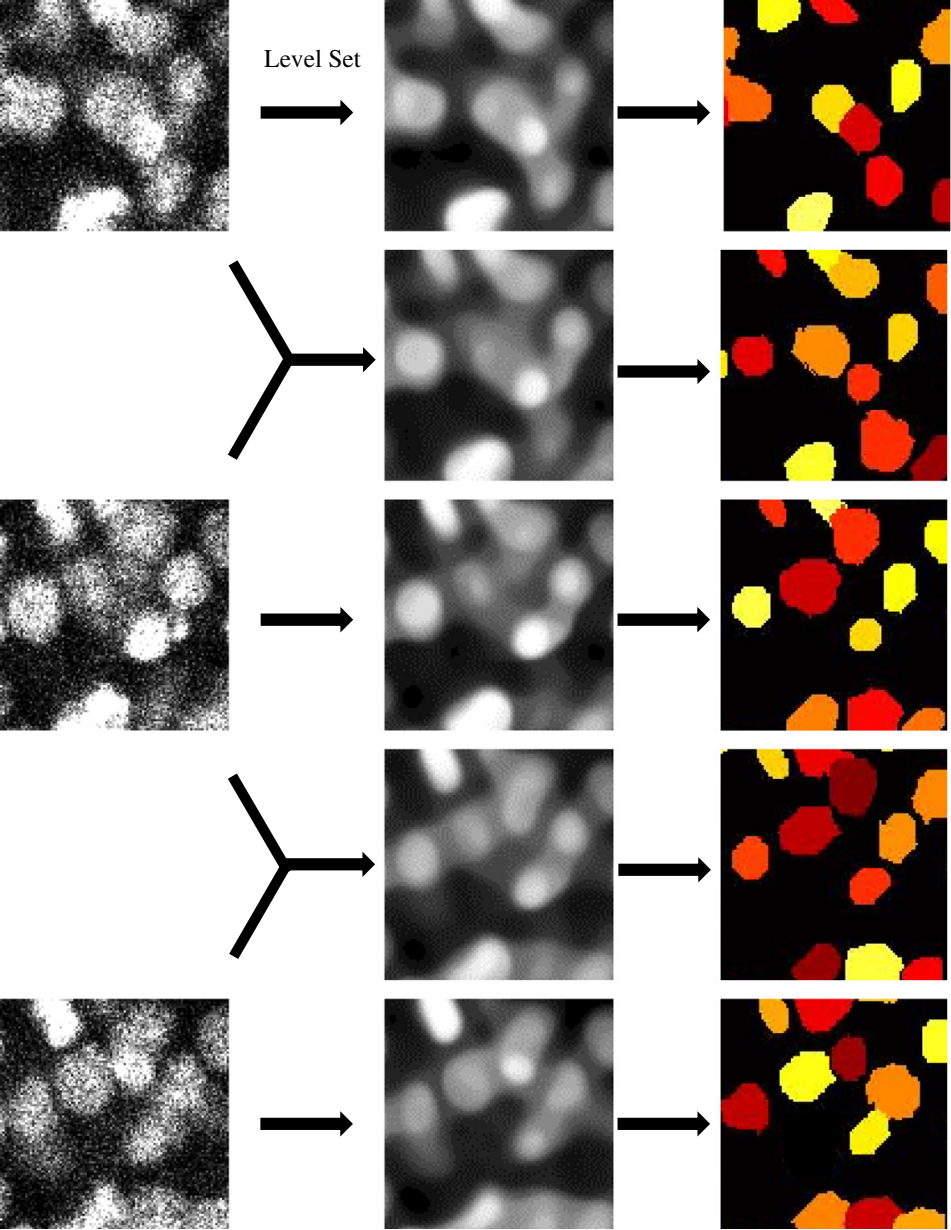
Level Set and 2D segmentation with region growing. The results are color-coded.

**Figure 3:**
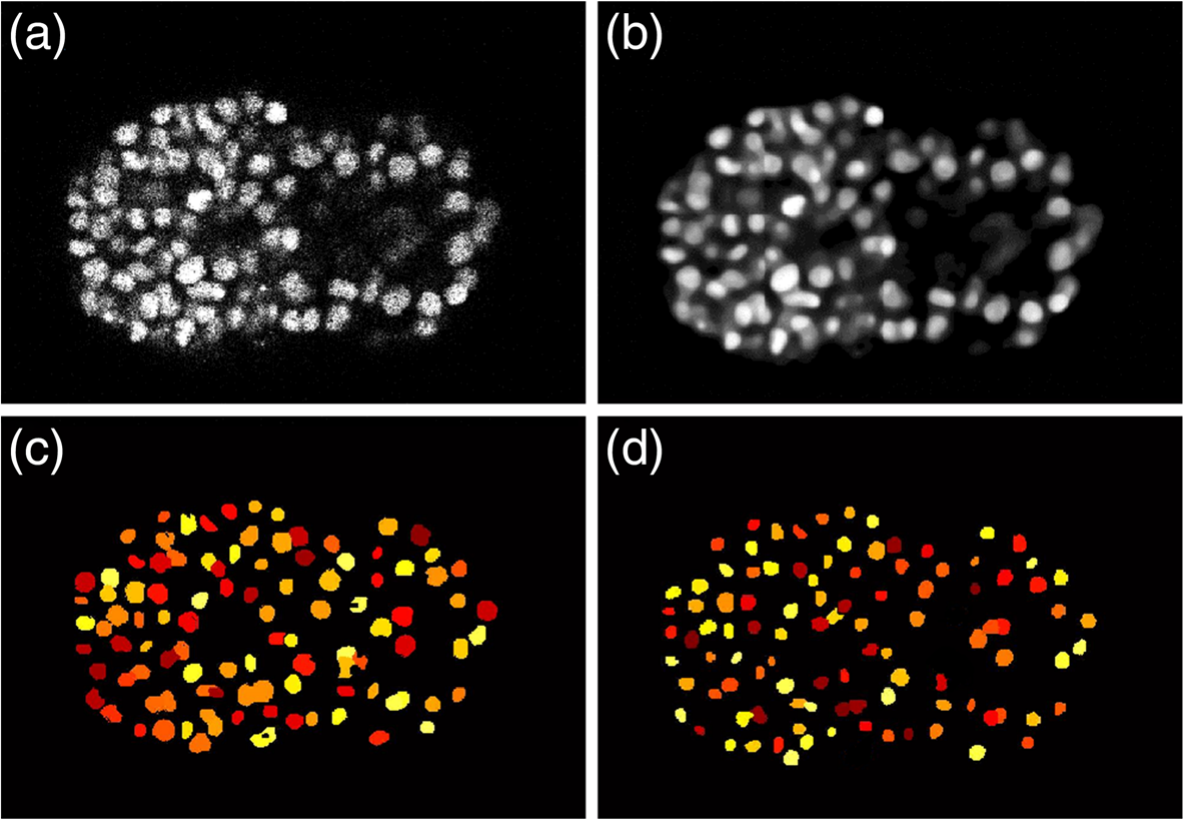
**Level set and 2D slice segmentation. (a)**. original image. **(b)**. image after level set. **(c)**. 2D slices by region growing. **(d)**. final result after image thinning.

The level-set method is a numerical technique for tracking interfaces and shapes, it can also be applied to smooth an image under the its curvature. Figure 2 shows the pre-processing method we use. As can be seen from the results shown in Figure 3, the level-set method can filter out noises existing in original images. It can also sharpen nuclei edges at the same time. After preprocessing with the level-set method, the images can be segmented with a region growing method. By using the level-set method we can obtain a predicted image between each pair of adjacent images, the total number of images in one single time point is increased from 41 to 81, thus we could generate more slices for nuclei detection.

We then test the new image series and compare with the original StarryNite. The results indicate that the number of errors generated by StarryNite has shown an appreciable decrease after level-set processing (350 cell stage). (Shown in Tables 2 and 3)

**Table 2:** **Error analysis of Matlab StarryNite**[9]

**Dataset**	**Segmentation errors**				**Tracing errors**	**Total**	**Total cell number**
	**False positives**	**False negatives**	**False diameters**	**Dislocations**			
130108PHA4p1	134	1145	488	124	540	2430	17103
130108NHR25p1	122	1205	513	168	478	2486	18723
Total	256	2350	1001	291	1018	4916	35826

130108PHA4p1 and 130108NHR25p1: 190–220 time points (1.5 minutes per time point).

**Table 3:** Error analysis of before and after level set processing

**Dataset**	**Segmentation errors**				**Tracing errors**	**Total**
	**False positives**	**False negatives**	**False diameters**	**Dislocations**		
130108NHR25p1 (before Level Set)	204	760	253	168	284	1669
130108NHR25p1 (after Level Set)	184	532	240	134	219	1309

#### Step2: 2D segmentation using the modified region growing method

In order to use the region growing method, the first and foremost step is to predetermine the initial ‘seed’ points, each of which will then grow into one 2D nuclei slice after being processed by a region growing algorithm. The method we use to search seed points is the 2D local maximization algorithm. Following our goal of obtaining precise segmentation of nucleus slices, we improve the design of the region growing algorithm in our nuclei segmentation system, so that it can perform well even under the condition in which the nuclei are crowded together. The traditional region growing algorithm is an iterative process, in which neighboring pixels of initial “seed points” are examined and one fixed threshold is used to determine whether the pixel neighbors should be added to a certain region. In our modified region growing method, the value of the threshold is determined by the distance between each pixel and seed point respectively, as a precaution against nuclei with lower expression values being overshadowed by brighter neighboring ones when they are overcrowded.

According to the nuclei diameter (1.8-7 microns) and the image resolution in the Z-axis (0.355 microns now, improved by Level Set), every nuclei will correspond to 4–18 2D nuclei slices, so even if some 2D nuclei slices of one nucleus cannot be recognized in some Z-planes, we can still identify them by the 2D local maximum points in the other Z-plane images. However, StarryNite/MatLab StarryNite uses only the method in which each 3D local maximum point is considered as one nucleus, therefore, one nucleus will be left out in the case where the corresponding 3D local maximum point is omitted. Thus it can be said in this sense that our method based on 2D nuclei slices is fault tolerant, which can reduce the false negative errors significantly.

#### Step3: Nuclei generation

**Figure 4:**
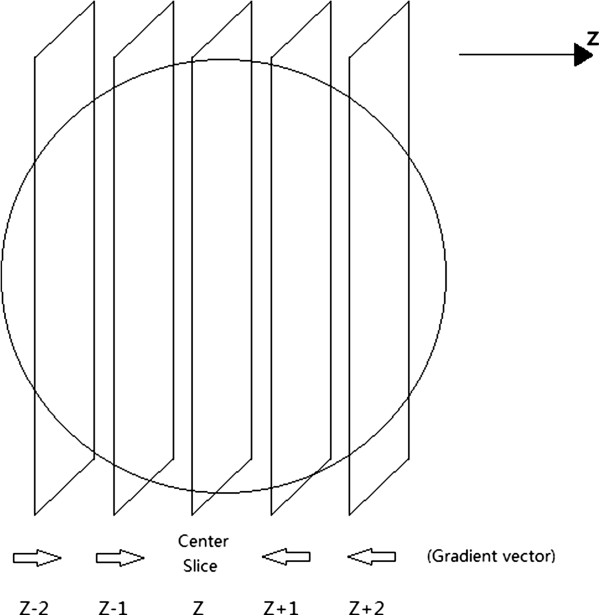
Method for center slice identification.

Santella et al. [9] also developed a nuclei segmentation method based on 2D slices, but their method still requires 3D local maximum points as indicators for nuclei determination. However, given the above analysis, the 3D local maximum point is not error free especially after the 350 cell stage. Hence, there are still many nuclei not being captured in the StarryNite/MatLab StarryNite output. Our method avoids this weakness of the 3D local maximization algorithm and identifies nuclei only by analyzing the features of the 2D slices. If one 2D slice is positioned in one nucleus center, this slice can be called a center slice, and each nucleus has only one center slice. Therefore after obtaining all the center slices at different time points, we will finally get the positions of all nuclei at different time points correspondingly. We use a modified gradient method to recognize all the center slices, as shown in Figure 4.

If one slice’s z-axial gradient of gray value conforms to the following equation, it will be chosen as one nuclei center slice.

(1) $$G_{Z+1}^{n}<0,$$

(2) $$G_{Z-1}^{n}<0,$$

(3) $$\left|G_{Z}^{n}\right|<\left|G_{Z‒1}^{n}\right|,$$

(4) $$\left|G_{Z}^{n}\right|<\left|G_{Z+1}^{n}\right|,$$

where $G_{Z}^{n}$ is the mean gradient of slice n on image Z, and $G_{Z‒1}^{n}$ is the mean gradient of slice n on image Z-1, the z-axial position of which is located just below image Z.

Due to the fact that the center slices contain data of the center position of each nucleus, we can work out which of the remaining slices are the constituent parts of one certain nucleus by calculating distances between the remaining slices and each center slice respectively. Each nucleus turns out to be composed of 4–18 slices in this case and information about exact position, radius, volume and brightness of every nucleus is achieved through this procedure.

#### Step4: Bi-directional prediction

After step 3, owing to the poor quality and low Z-axial resolution of images, some nuclei segmentation errors especially the false negative errors surface inevitably if we do nuclei segmentation based only on images of one single time point. During the process of embryogenesis, every new nucleus in the lineage tree comes from cell division. These new nuclei will divide into other new nuclei or die, in other words, the same nuclei may not be captured via single-time images. Further, the shift distance of every nucleus between two adjacent time points is very short, and the nuclei size will not change much within short-time intervals, so we can refer to the image before or after the current time point to forecast a nucleus in order to avoid the failure of capturing it at one single time point. This process effectively decreases the emergence of false negative errors. Considering the possibility that one nucleus may divide into two nuclei during embryogenesis, a bi-directional prediction instead of only a forward or backward prediction is necessary and effective in nuclei prediction.

In order to achieve this bi-directional prediction, a score function is established to compute the degree of dissimilarity between two nuclei at two adjacent time points. In our score function, two aspects are considered: shift distance and nuclei volume.

(5) $$DS_{\mathrm{AB}}=\frac{d}{0.5\times \left(r_{A}+r_{B}\right)}+\frac{\left|\frac{V_{A}}{V_{B}}-1\right|}{1.5},$$

where A and B are the symbolic representation of the nuclei; *DS*_*AE*_ is the the dissimilarity degree between nuclei A and B; *d* is the shift distance between A and B; *r*_*A*_*, r*_*B*_ are the radii of A and B respectively; and *V*_*A*_ and *V*_*B*_ are the volume of A and B respectively.

**Figure 5:**
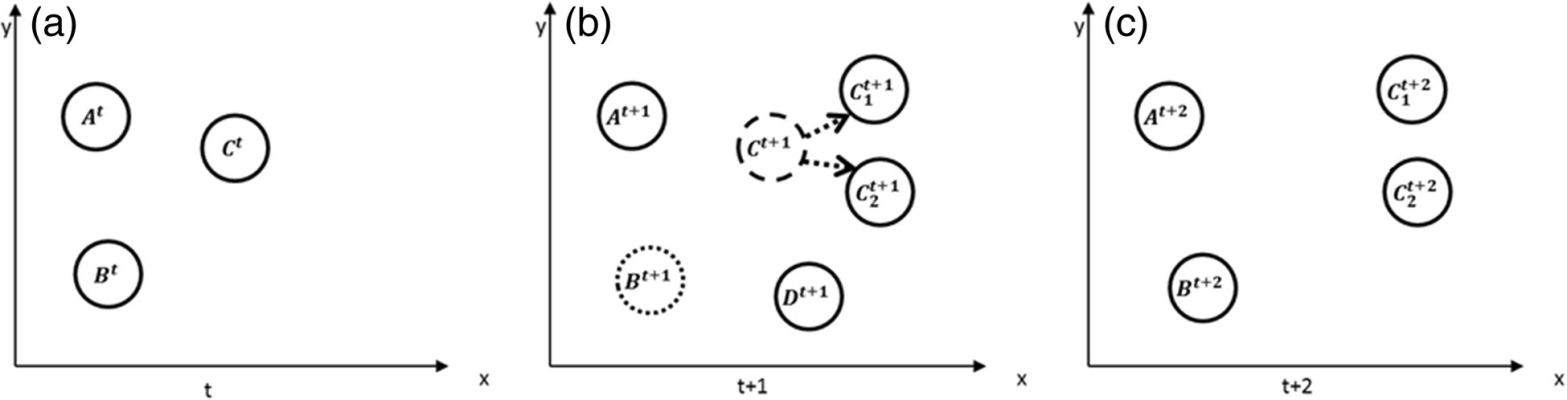
**Bi-directional prediction. (a) ***B*^*t*+1^ can be predicted by *B*^*t*^ and *B*^*t*+2^. **(b) ***D*^*t*+2^ is a false positive error. **(c) **$C_{1}^{t+1}$ and $C_{2}^{t+1}$ are true positive nuclei.

If *DS*_*AE*_ is less than 1.5, A and B are likely to be the same nuclei at different time points. Figure 5 gives an illustration of this case, where no nuclei *B*^*t*+1^ exists in t + 1 time point image, but according to images before and after, $DS_{B^{t}B^{t+2}}$ is less than 1.5, so the missed nuclei *B*^*t*+1^ can still be predicted. Besides, if a nucleus captured by a single time point does not exist at the adjacent time points, we can come to the conclusion that this nucleus is a false positive. An example, nuclei *D*^*t+*1^ at time point t + 1, is shown in Figure 5.

Both false positive errors and false negative errors can be decreased significantly through our bi-directional prediction method. Bi-directional prediction is capable of dealing with some problems that cannot be solved by simply improving image segmentation, and this is the most significant innovation of our method.

#### Step5: Nuclei filtering

Through the above processing, all the possible nuclei for each time point are recognized and saved in one nuclei list. However, at several consecutive time points, some false positive errors which cannot be eliminated by our bi-directional prediction method, still remain, so we need a filter to deal with this kind of error.

**Figure 6:**
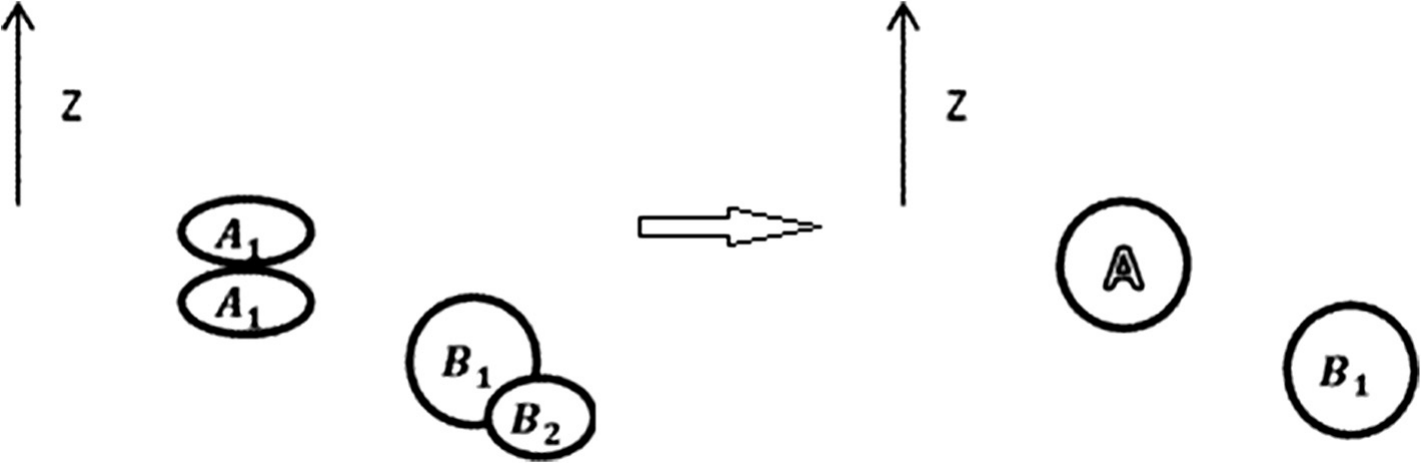
**Two kinds of false positive errors. (a) ***A*_1_ and *A*_2_ will merge into A. **(b) ***B*_2_ will be deleted.

There are two categories of false positive errors (see Figure 6). First, the nucleus is actually part of another nucleus; second, the nucleus does not really exist. To distinguish these two kinds of errors, we calculate the distances between every pair of nuclei, and then form a judgement based on the following principles.

If there are two nuclei A and B, and their coordinates are (*x*_*A*_*, y*_*A*_*, z*_*A*_) and (*x*_*B*_*, y*_*B*_*, z*_*B*_) respectively, *d*_*AE*_ and *D*_*AE*_ are defined as

(6) $$D_{\mathrm{AB}}=\sqrt{{\left(x_{A}-x_{B}\right)}^{2}+{\left(y_{A}-y_{B}\right)}^{2}+z_{A}-{z_{B}}^{2}},$$

(7) $$d_{\mathrm{AB}}=\sqrt{{\left(x_{A}-x_{B}\right)}^{2}{\left(y_{A}-y_{B}\right)}^{2}},$$

We consider that nucleus A and B are actually part of each other if nuclei A and B satisfy the following relationship, where *R*_*A*_ and *R*_*B*_ are the radii of nuclei A and B respectively, and then we merge A and B into one new nucleus:

(8) $$D_{\mathrm{AB}}<a\times \left(R_{A}+R_{B}\right),\left(0.6<a<0.9\right),$$

(9) $$d_{\mathrm{AB}}<\beta \times \left(R_{A}+R_{B}\right),\left(0.35<\beta <0.6\right)$$

We consider either nucleus A and B does not really exist, if nuclei A and B satisfy the following relationship

(10) $$D_{\mathrm{AB}}<a\times \left(R_{A}+R_{B}\right),\left(0.6<a<0.9\right)$$

(11) $$d_{\mathrm{AB}}>\beta \times \left(R_{A}+R_{B}\right),\left(0.35<\beta <0.6\right)$$

Then we select nucleus A or B to be deleted based on their volume and gray values.

Finally, an optimal nuclei list is obtained, representing the expected properties including nuclei ID, 3D center coordinate, nucleus volume, radius, 2D slice and gray value. With this list we can generate the information for lineage tracing. In fact for the existing tracing method, the necessary information is the 3D coordinate of the nuclei center and gray value. Other very important information can be used to improve the tracing algorithm in StarryNite.

### Experiment results

We used our method on data sets obtained from our Leica SP5 microscope. [Additional files 2, 3, and 4] We also applied our nuclei segmentation results to the generation of a lineage tree with the tracing algorithm in StarryNite. The performance of the nuclei segmentation method can be evaluated by the following aspects: Nuclei number, accuracy rate and editing time needed for building a lineage tree.

#### Nuclei number

**Figure 7:**
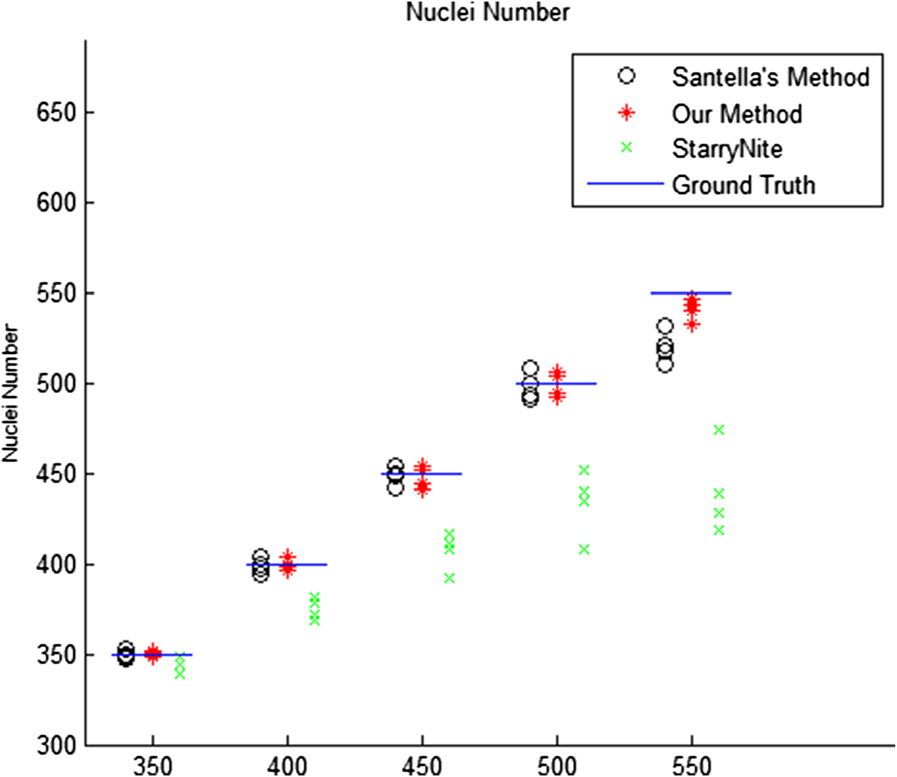
Nuclei number after segmentation.

The maximum number of nuclei that can be detected (before tracing) by StarryNite [4] and Matlab StarryNite [9] for a single time point is about 450 and 550, respectively. For Matlab StarryNite, after the 500 cell stage, the false negative errors begin to appear in large numbers. At the 500–600 cell stage, nuclei segmentation of StarryNite will miss 20–60 nuclei in every time point, thereby the error rate at the 500–600 cell stage will be 4%-12%, leading to omissions of large numbers of nuclei in the lineage tree built by the tracing part even without any tracing errors. It makes it almost impossible for further editing after the 500 cell stage. However, in our method, as the nuclei number data shows in Figure 7, a considerable advance in nuclei number can be clearly observed at the 500–600 cell stage.

#### Error rate

**Figure 8:**
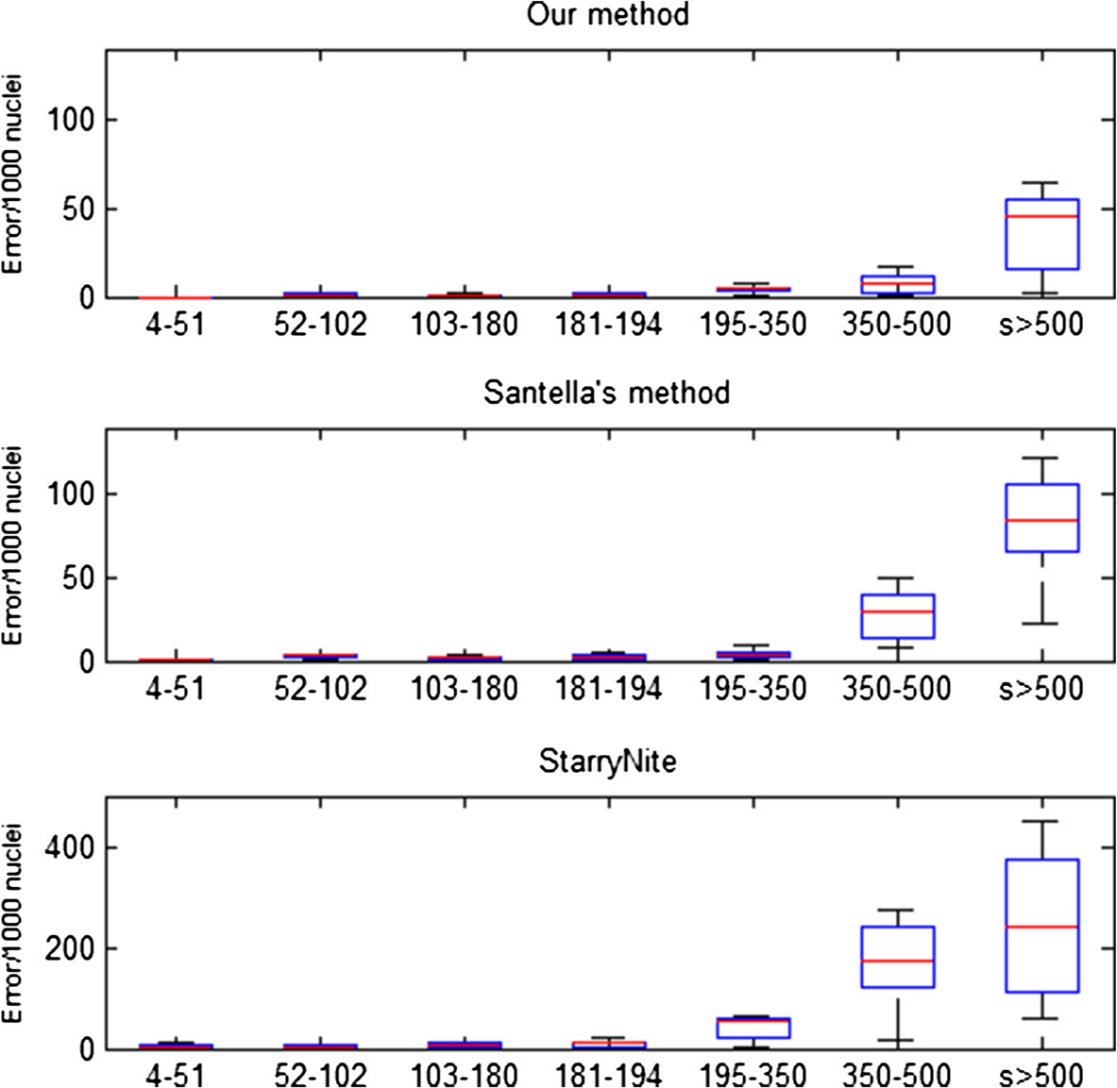
Nuclei segmentation accuracy rate (4–550 cell stage).

**Figure 9:**
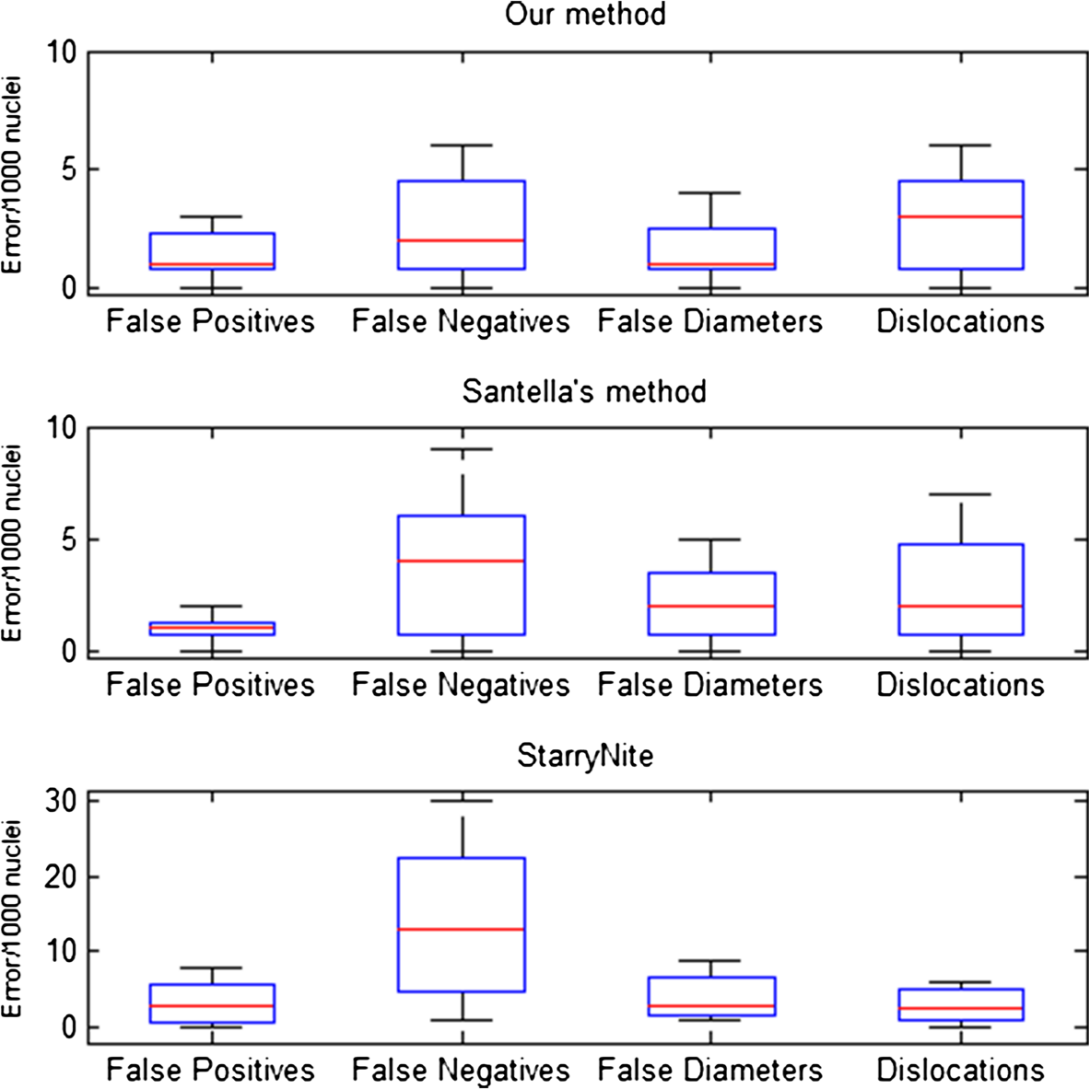
Nuclei segmentation accuracy rate (195–350 cell stage).

**Figure 10:**
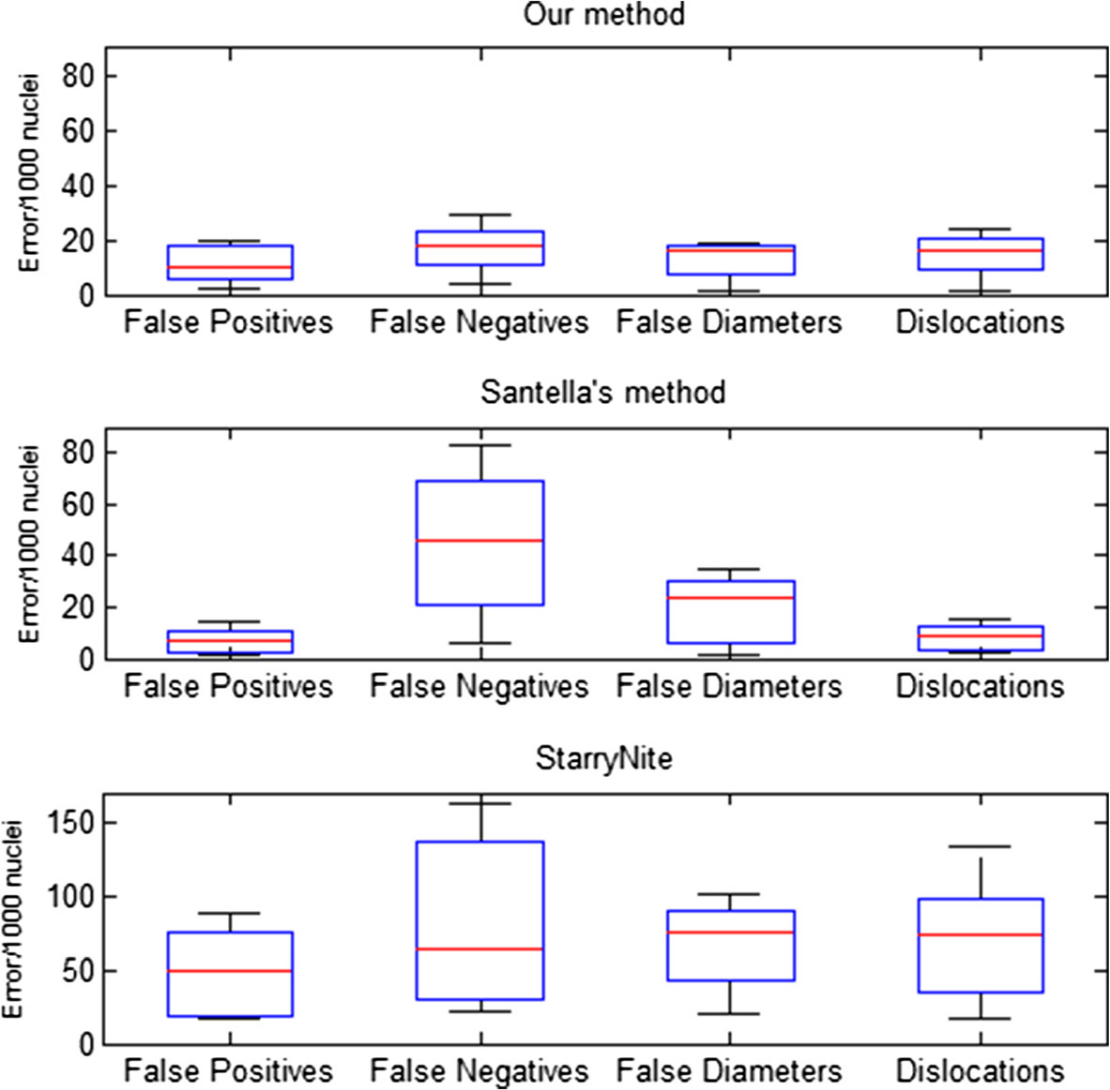
Nuclei segmentation accuracy rate (450–550 cell stage).

The number of nuclei is just a coarse evaluation index of performance of a nuclei segmentation system, because recognition errors of nuclei may still exist as interfering factors, while a leap in accuracy rate can directly reflect the application value of our method. As previously mentioned, the main error types caused by nuclei segmentation are false positives and false negatives. It can be seen in Figures 8, 9 and 10 that our nuclei segmentation is much better than Matlab StarryNite (Santella’s method) in at the 450–550 cell stage when tested on our data set. Furthermore, at the 500 cell stage, a very low error rate (<4%) is still maintained as an assurance for the stability of the tracing result by using our nuclei segmentation system. The overall error rate (4–550 cell stage) is less than 1%.

All our data sets are sampled with 90 seconds time resolution, but a better result will be reached if the time resolution is increased to 60 seconds as in the image protocol in Aydin [10]. This is because the nuclei will become shorter as the time resolution is increased from 90 seconds to 60 seconds, which will greatly reduce the difficulty in the following bi-directional prediction.

#### Editing time

Editing time needed for one correct lineage tree is another important performance criterion. In Bao [4], StarryNite requires 4–10 hours to edit the tree at the 350 cell stage, and due to the aforementioned maximum-number limit of nuclei by the nuclei segmentation part in StarryNite, it is quite hard to edit the lineage tree beyond the 350 cell stage. One lineage tree, which was edited to the 450 cell stage using StarryNite, required more than one week of manual editing [20]. Santella’s 2D blob-slice method performed much better than StarryNite, Richards [11] used Santella’s method and could reach the 550 cell stage with 8–16 hours manually editing. Their images have a double high resolution in the Z direction, that is there are 60 images in every time point, nearly twice the number of images used in our case. As the number of images double, the storage space required for image and computation time also doubles as a consequence. We performed our method on data sets 130108NHR25p1 and 130108PHA4p1, which requires only 30 minutes for a 350 cells lineage tree, and eight hours for a 450 cell lineage tree.

## Discussion

There have been several methods for automatic cell lineage tree generation. StarryNite [4] together with a tool named AceTree [1] provided an efficient system for cell recognition and annotation. Santella [9] and Richard [11] improved the nuclei segmentation with a different method. Santella [9] revised the nuclei segmentation with a 2D blob-slice method, and Richard [11] advanced the output of the tracing procedure by increasing the image resolution of the Z-axis with a new type of resonance-scanning microscopy. Due to poor image quality and the rapid multiplying of nuclei after the 350 cell stage, there are substantial nuclei segmentation errors, especially false negative ones, causing large numbers of unavoidable follow-up errors and long time manual editing of the cell lineage. Therefore a bi-directional prediction technique was developed by us as an efficient way to alleviate these issues. At the same time, other methods such as level-set, region growing and a modified gradient algorithm has also been adopted for the purpose of improving our nuclei segmentation system.

In our work, instead of using single time point images for nuclei segmentation, we take advantage of the bi-directional prediction technique, analyzing the images before and after the current time point to improve nuclei identification. As the interval between two adjacent sampling times is only 50–90 seconds, the coordinates, size, and expression value of the same nucleus in two adjacent time points remain nearly unchanged. This feature significantly reduces the segmentation errors in several continuous time points. One omitted nucleus could certainly be discovered from nuclei segmentation in another time point. We also apply level-set, 2D region growing and the gradient method to our whole nuclei segmentation system. First, the level-set method boosts the image resolution in the Z-axis, and ensures the integrity of the edge information by smoothing the image under the curvature. Moreover, noises existing in the images also get reduced by the level-set method. Second, our 2D region growing method can achieve an accurate 2D slice segmentation. It is mainly because the threshold for our region growing method varies inversely with the size of the nuclei slice, which guarantees that the slices will not be eclipsed by a brighter neighbor. Third, the modified gradient method is an effective way to search for the center slices, each of which represents one exclusive nucleus. As demonstrated in experiment results, when the error rate is low, the outcome of our nuclei segmentation method is much better in nuclei reorganization. But due to the existence of errors caused by the tracing part of StarryNite, it is still impossible to get a perfectly accurate lineage without manual editing. Future research may include work on improving the performance of the tracing algorithm. In fact, the weakest part of the tracing algorithm lies in the cell-division detection, as the algorithm is mainly based on the assumption that the parent cell is located at the center position of two daughter cells. However, at the 350 cell stage, a satisfactory result cannot be achieved via the present day cell-division detection algorithm, since the space distribution pattern of a cell becomes more complicated. At the same time, nuclei segmentation errors are common, which indicates that there is still room for much improvement of the StarryNite method through the optimization of the tracing algorithm.

## Conclusion

In this paper, we present a novel nuclei segmentation system which can be used for automatic cell lineage tracing. Several new designs are introduced using our method, including level-set, region-growing, gradient tracing and bi-directional prediction. Among these techniques, the bi-directional prediction, which is capable of detecting nuclei based on backward and forward information, provides a major advance in the nuclei segmentation algorithm. The results using our image data also demonstrate that the proposed algorithm is accurate and practical.

There are still two aspects which need to be noted. First, our level-set preprocessing is time-consuming. This problem can be solved in the future by using more efficient algorithms and more powerful computers. Second, at a very late stage (at the 500 cells stage), some small and non-spherical nuclei may appear, which are hard to distinguish with the limitations in the current image resolution.

Subsequent research may focus on the combination of nuclei segmentation and tracing procedure. A real-time lineage tracing system may then be achieved with this kind of architecture.

## Methods

### Date sets

The data sets we used are obtained from a Leica SP5 confocal microscope using Hybrid detector, and images were taken every 90 seconds with the same x and y resolution as described previously (ref). The data sets contain 41 image planes in every time point, the distance between two adjacent images planes is 0.71 microns (Additional file 1: Table S1).

### Level-set

The level set method (LSM) is a kind of numerical technique which can be used for tracking interfaces and shapes. The advantage of the level set method is that one can perform numerical computations involving curves and surfaces on a fixed Cartesian grid without having to parameterize these objects (this is called the Eulerian approach) [21]. The level set method provides us with an easy way to smooth the 3D time-lapse image of *C. elegans* without losing important image details. The method has laid a foundation for 2D slice segmentation by region growing.

We have used a Matlab toolbox from http://barissumengen.com/level_set_methods/. The iteration number is 6, and at the 500 cell stage, the iteration number is 4.

### Region growing

Region growing is a kind of pixel-based image segmentation method [22]. This approach to segmentation examines neighboring pixels of initial “seed points” and determines whether the pixel neighbors should be added to the region depending on a region membership criterion. In our method, this kind of growing process is iterated on, and every seed point will grow into a nucleus slice. These nuclei slices will be used in nuclei recognition.

So the first step in region growing is to select a set of seed points. We use the 2D local maximum points as the seed points. The initial region begins at the exact location of the 2D local maximum points. We assume that there are three pixels, A, B and S, pixel A and S are members of region R, and pixel S is the seed point of region R. Pixel B does not belong to region R but B is a neighbor pixel of A. If pixel A and B satisfy the following relationship, pixel B will be added to region R.

(12) $$\left|G_{A}-G_{B}\right|<4\left(\frac{G_{A}}{150}+1\right)\left[1-{\left(\frac{d_{\mathrm{BS}}}{r}\right)}^{2}\right],$$

*G*_*A*_ and *G*_*B*_ is the gray value of pixel A and B respectively, *r* is the max radius of all nuclei in previous time points, *d*_*BS*_ is the distance between B and S.

## Competing interests

The authors declare that they have no competing interests.

## Authors’ contributions

LC carried out data analysis and drafted the paper. LLC, ZZ and HY initiated the image processing part of the project and helped with the writing of the paper. ZZ’s group obtained the image data. All authors read and approved the final manuscript.

## Supplementary Material

Additional file 1 Details of image, error examples and supplemental figures. Click here for file

Additional file 2 Experiments results and the parameter files we used. Click here for file

Additional file 3 The program based our method and a sample manual. Click here for file

Additional file 4 The program for pre-processing and a sample manual. Click here for file
