# Supplementary material for: A novel cell nuclei segmentation method for 3D C. elegans embryonic time-lapse images
Source: BMC Bioinformatics. 2013 Nov 19;14:328. doi: 10.1186/1471-2105-14-328 (PMC3903074; doi:10.1186/1471-2105-14-328)

**Additional Tables, Figures and Methods**

**Additional Table 1 Image sampling resolution for each test data set.**

|  | *C. elegans*  *(our 1)* | *C. elegans*  *(our 2)* | *C. elegans*  *(published, Bao, 2006)* |
| --- | --- | --- | --- |
| X,Y res (µm) | 0.25 | 0.25 | ~0.254 |
| Z res (µm) | 1 | 0.71 | ~1 |
| Voxel aspect ratio | 4 | 2.84 | ~3.94 |
| Nuclear diameter (µm) | ~5-3 | ~5-3 | ~5-3 |
| Nuclear separation (µm) | ~2-.4 | ~2-.4 | ~2-.4 |

**Additional Table 2** **Computational load of processing a full image volume.**

|  | *C. elegans*  *(our 1)* | *C. elegans*  *(our 2)* | *C. elegans (published, Bao 2006)* |
| --- | --- | --- | --- |
| Volume dimensions (pixels) | 712x512x31  (11.3 mega pixels) | 712x512x41  (14.94 mega pixels) | 708x512x35 (12.6 mega pixels) |
| Temporal sampling (minutes between samples) | 1.5 | 1.5 | 1 |
| Total time points | 200 | 200 | 99 |
| Number of detected cells | 550 | 550 | 90 |

**Additional Figure 1 Error examples.**


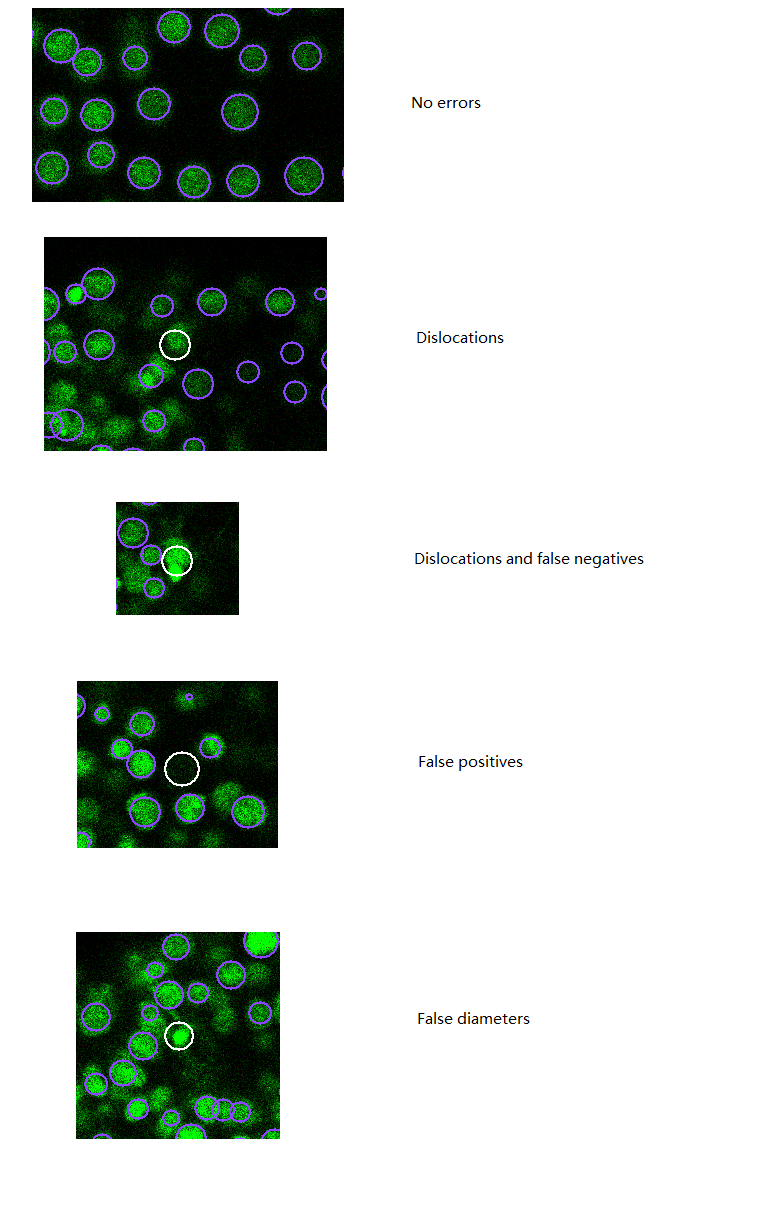

Supplement: Additional file 1 — Details of image, error examples and supplemental figures. [file 1471-2105-14-328-S1.docx]
