# Supplementary figures and images for: A novel cell nuclei segmentation method for 3D C. elegans embryonic time-lapse images
Source: BMC Bioinformatics. 2013 Nov 19;14:328. doi: 10.1186/1471-2105-14-328 (PMC3903074; doi:10.1186/1471-2105-14-328)

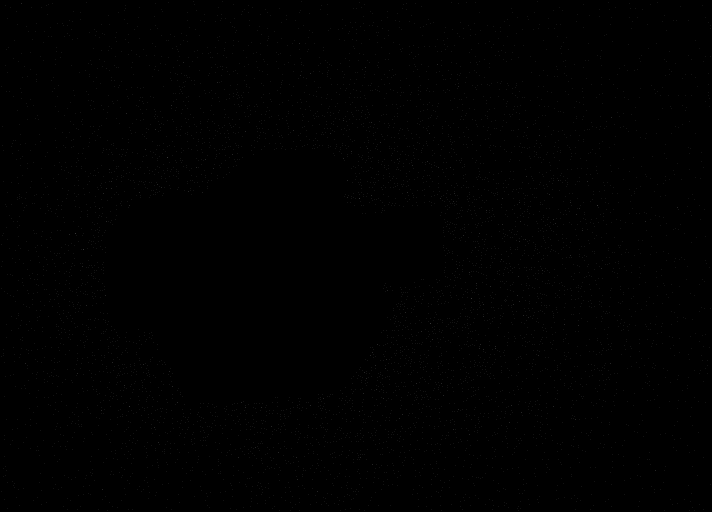

Supplement: Additional file 3 — The program based our method and a sample manual. [file 1471-2105-14-328-S3.zip › Additional_file3/processed_image/130108NHR25p1_L1-t193-p01.tif]

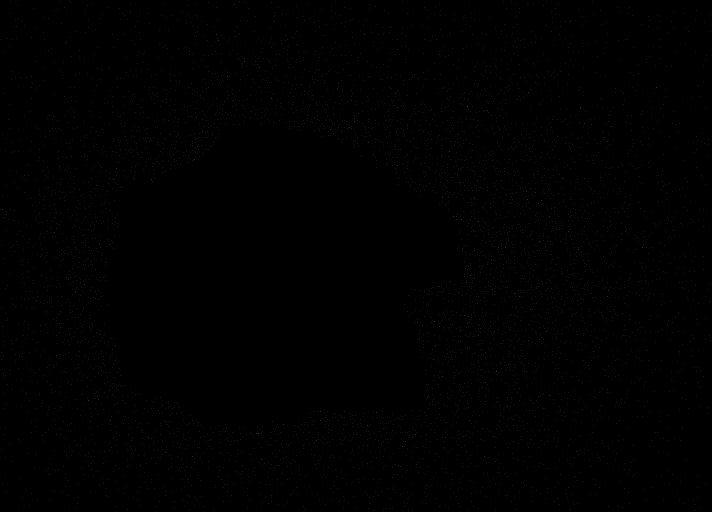

Supplement: Additional file 3 — The program based our method and a sample manual. [file 1471-2105-14-328-S3.zip › Additional_file3/processed_image/130108NHR25p1_L1-t193-p02.tif]

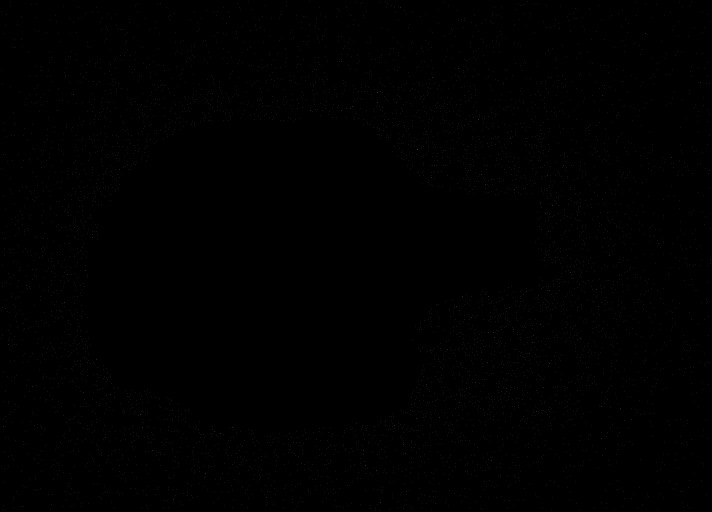

Supplement: Additional file 3 — The program based our method and a sample manual. [file 1471-2105-14-328-S3.zip › Additional_file3/processed_image/130108NHR25p1_L1-t193-p03.tif]

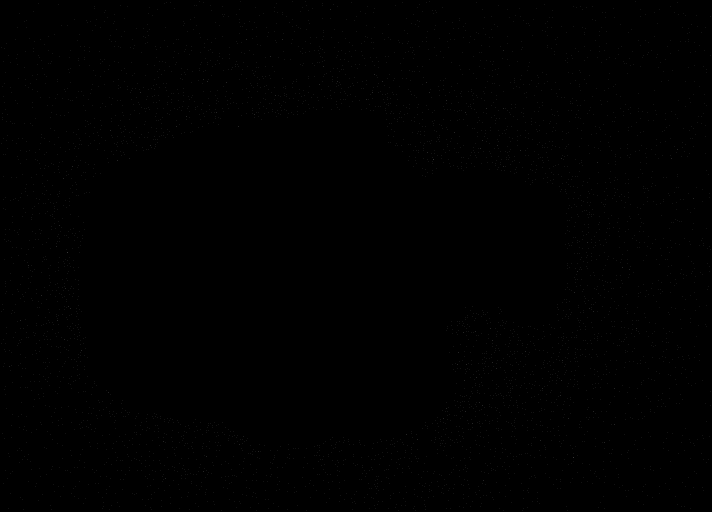

Supplement: Additional file 3 — The program based our method and a sample manual. [file 1471-2105-14-328-S3.zip › Additional_file3/processed_image/130108NHR25p1_L1-t193-p04.tif]

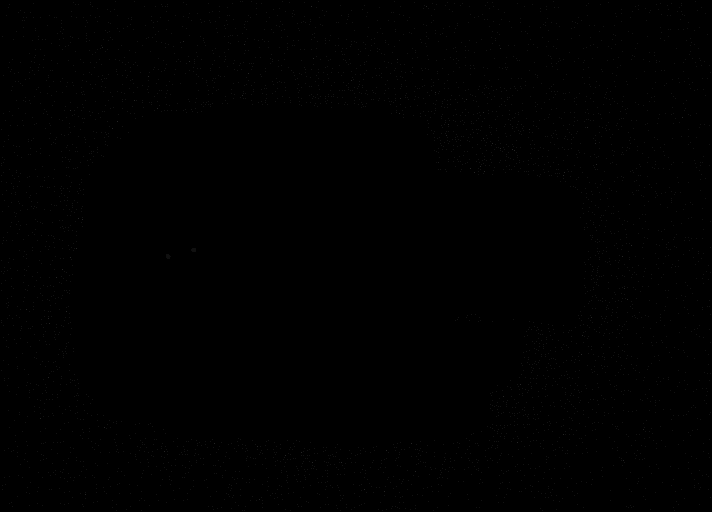

Supplement: Additional file 3 — The program based our method and a sample manual. [file 1471-2105-14-328-S3.zip › Additional_file3/processed_image/130108NHR25p1_L1-t193-p05.tif]

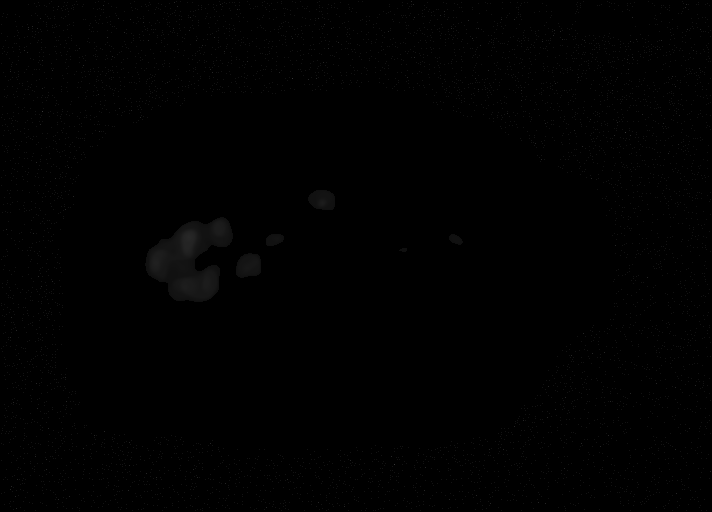

Supplement: Additional file 3 — The program based our method and a sample manual. [file 1471-2105-14-328-S3.zip › Additional_file3/processed_image/130108NHR25p1_L1-t193-p06.tif]

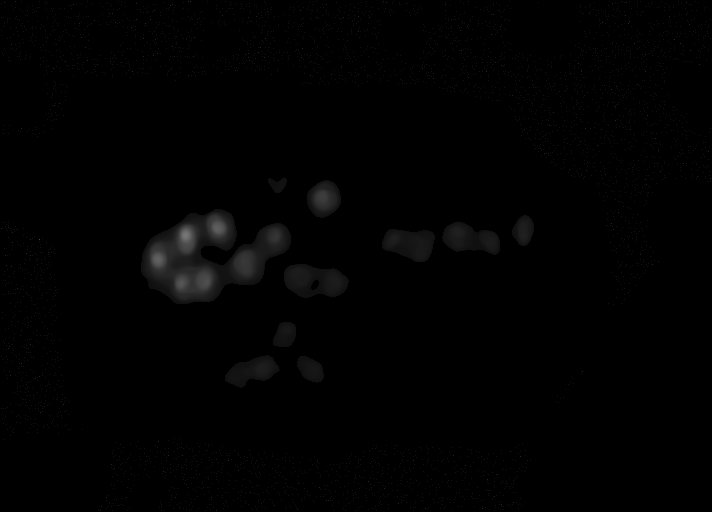

Supplement: Additional file 3 — The program based our method and a sample manual. [file 1471-2105-14-328-S3.zip › Additional_file3/processed_image/130108NHR25p1_L1-t193-p07.tif]

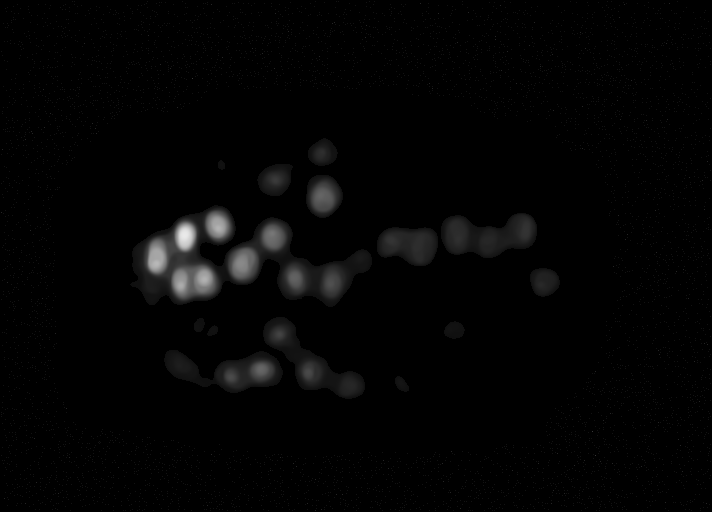

Supplement: Additional file 3 — The program based our method and a sample manual. [file 1471-2105-14-328-S3.zip › Additional_file3/processed_image/130108NHR25p1_L1-t193-p08.tif]

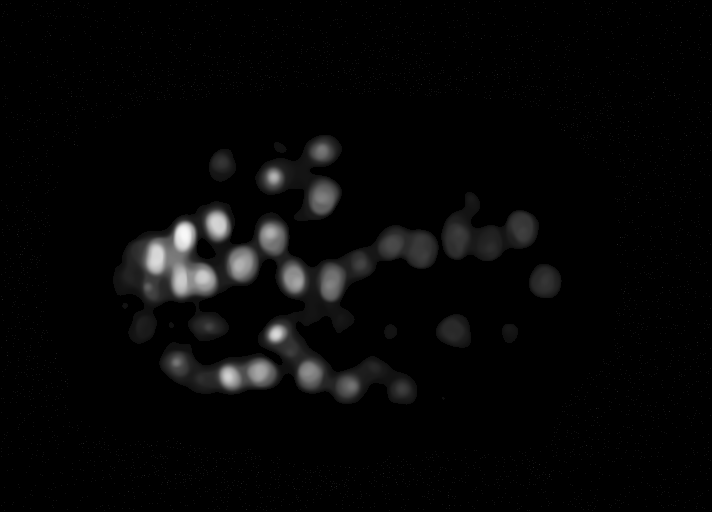

Supplement: Additional file 3 — The program based our method and a sample manual. [file 1471-2105-14-328-S3.zip › Additional_file3/processed_image/130108NHR25p1_L1-t193-p09.tif]

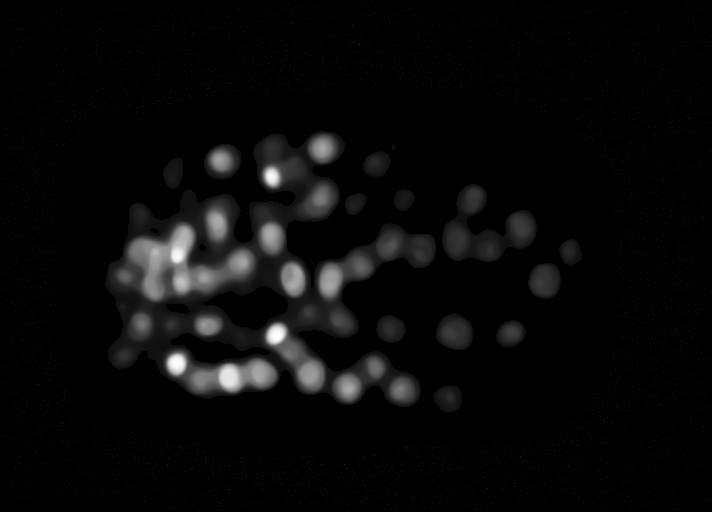

Supplement: Additional file 3 — The program based our method and a sample manual. [file 1471-2105-14-328-S3.zip › Additional_file3/processed_image/130108NHR25p1_L1-t193-p10.tif]

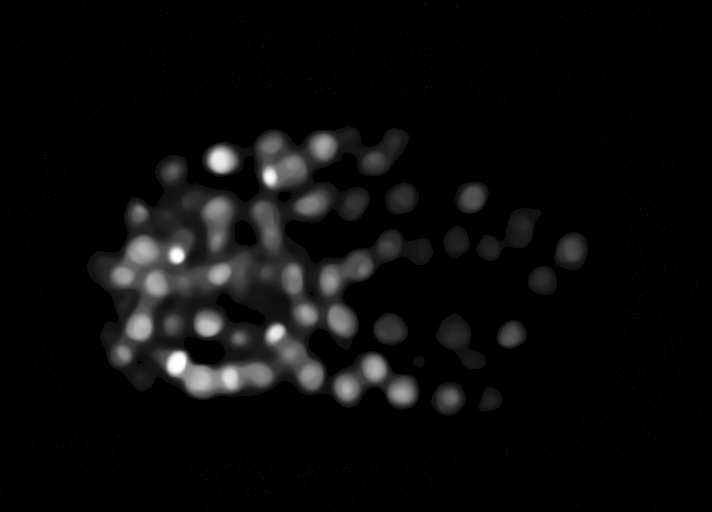

Supplement: Additional file 3 — The program based our method and a sample manual. [file 1471-2105-14-328-S3.zip › Additional_file3/processed_image/130108NHR25p1_L1-t193-p11.tif]

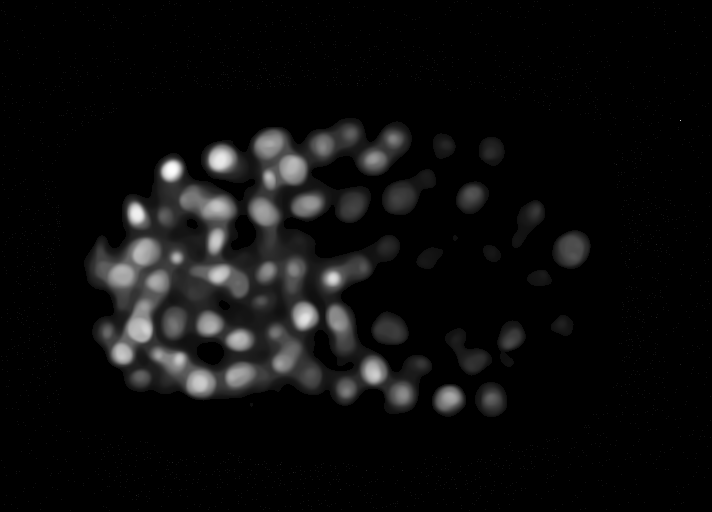

Supplement: Additional file 3 — The program based our method and a sample manual. [file 1471-2105-14-328-S3.zip › Additional_file3/processed_image/130108NHR25p1_L1-t193-p12.tif]

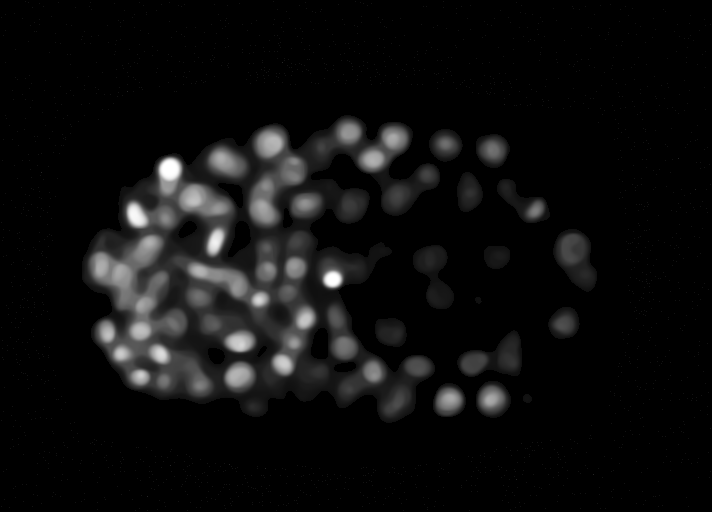

Supplement: Additional file 3 — The program based our method and a sample manual. [file 1471-2105-14-328-S3.zip › Additional_file3/processed_image/130108NHR25p1_L1-t193-p13.tif]

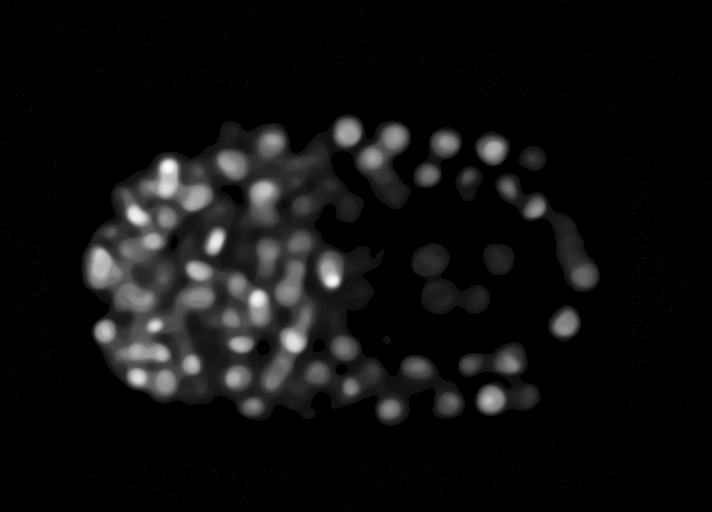

Supplement: Additional file 3 — The program based our method and a sample manual. [file 1471-2105-14-328-S3.zip › Additional_file3/processed_image/130108NHR25p1_L1-t193-p14.tif]

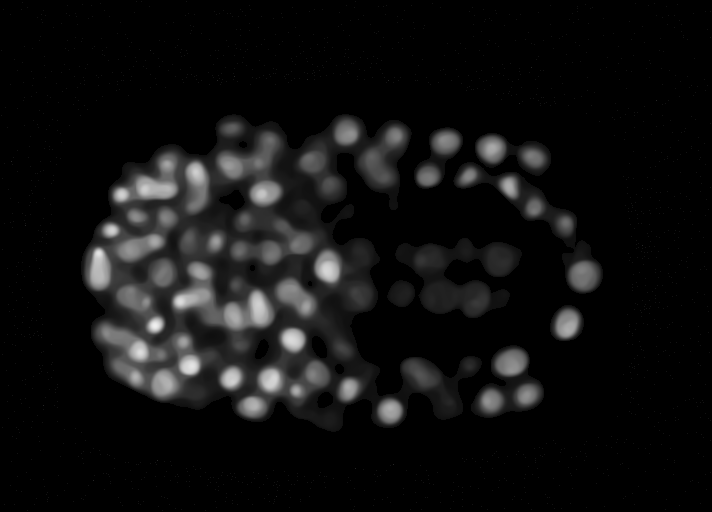

Supplement: Additional file 3 — The program based our method and a sample manual. [file 1471-2105-14-328-S3.zip › Additional_file3/processed_image/130108NHR25p1_L1-t193-p15.tif]

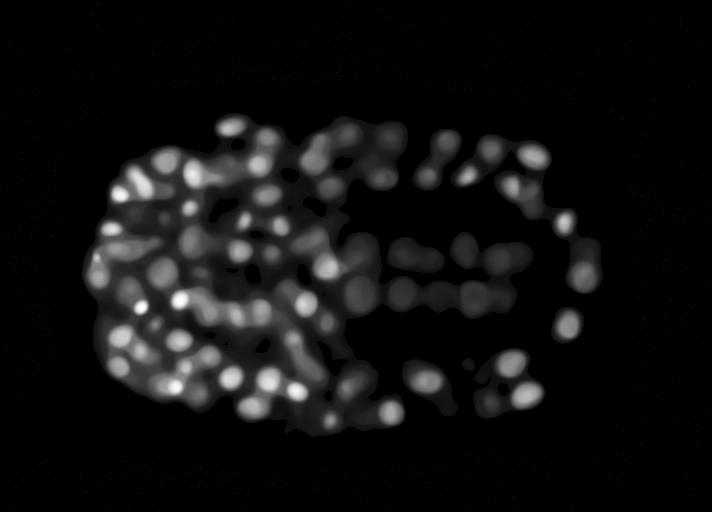

Supplement: Additional file 3 — The program based our method and a sample manual. [file 1471-2105-14-328-S3.zip › Additional_file3/processed_image/130108NHR25p1_L1-t193-p16.tif]

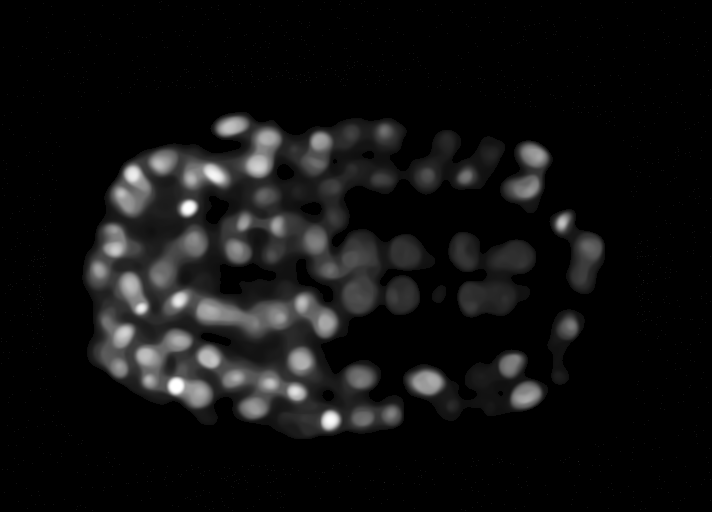

Supplement: Additional file 3 — The program based our method and a sample manual. [file 1471-2105-14-328-S3.zip › Additional_file3/processed_image/130108NHR25p1_L1-t193-p17.tif]

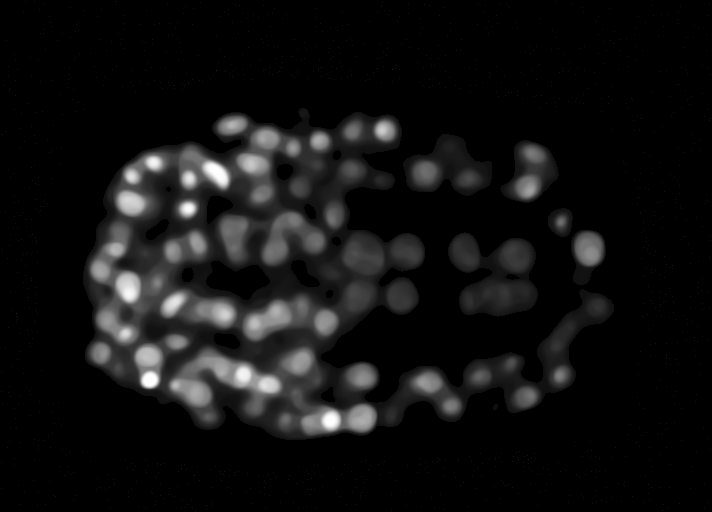

Supplement: Additional file 3 — The program based our method and a sample manual. [file 1471-2105-14-328-S3.zip › Additional_file3/processed_image/130108NHR25p1_L1-t193-p18.tif]

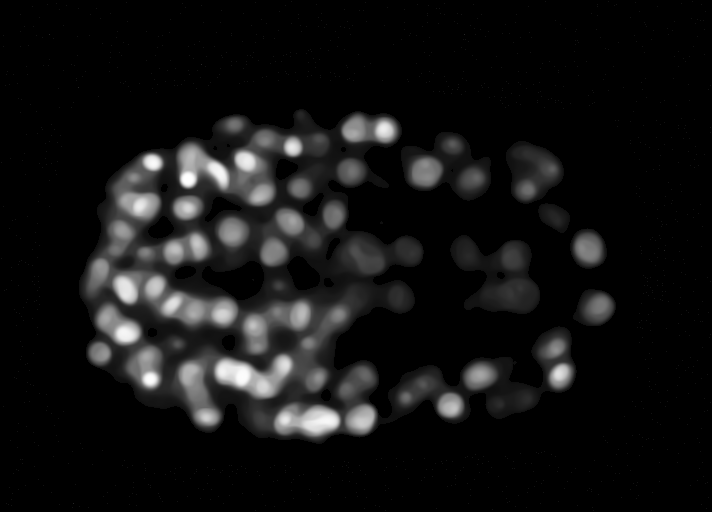

Supplement: Additional file 3 — The program based our method and a sample manual. [file 1471-2105-14-328-S3.zip › Additional_file3/processed_image/130108NHR25p1_L1-t193-p19.tif]

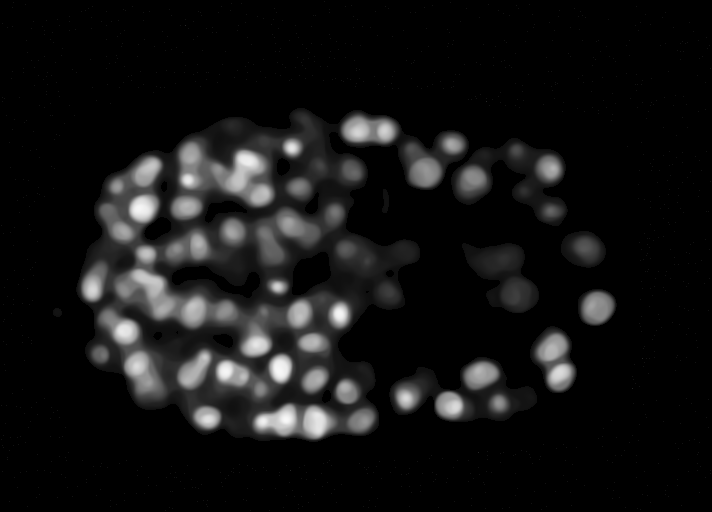

Supplement: Additional file 3 — The program based our method and a sample manual. [file 1471-2105-14-328-S3.zip › Additional_file3/processed_image/130108NHR25p1_L1-t193-p20.tif]

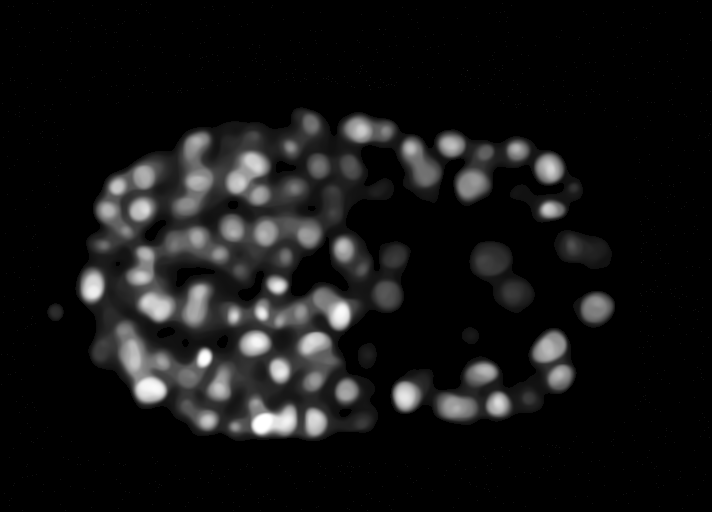

Supplement: Additional file 3 — The program based our method and a sample manual. [file 1471-2105-14-328-S3.zip › Additional_file3/processed_image/130108NHR25p1_L1-t193-p21.tif]

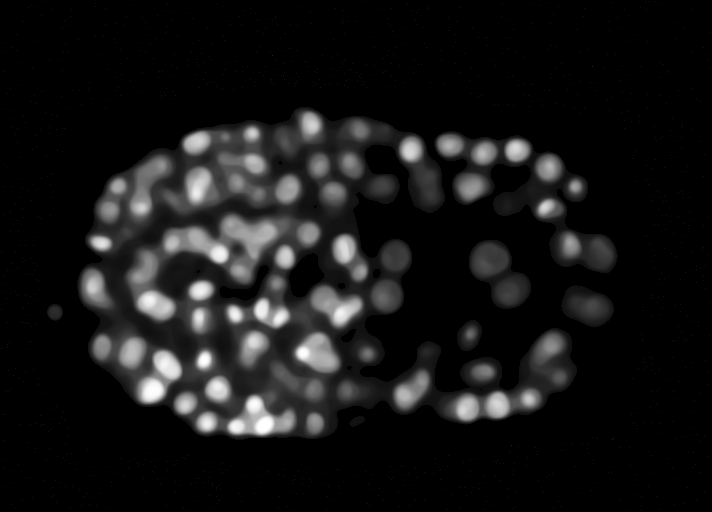

Supplement: Additional file 3 — The program based our method and a sample manual. [file 1471-2105-14-328-S3.zip › Additional_file3/processed_image/130108NHR25p1_L1-t193-p22.tif]

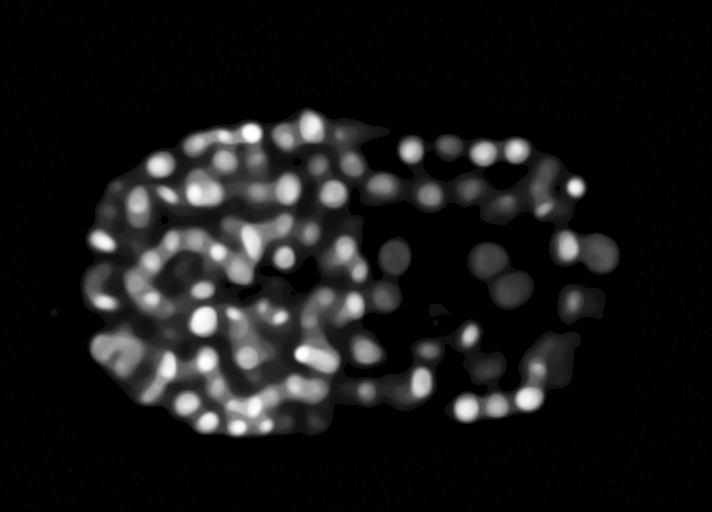

Supplement: Additional file 3 — The program based our method and a sample manual. [file 1471-2105-14-328-S3.zip › Additional_file3/processed_image/130108NHR25p1_L1-t193-p23.tif]

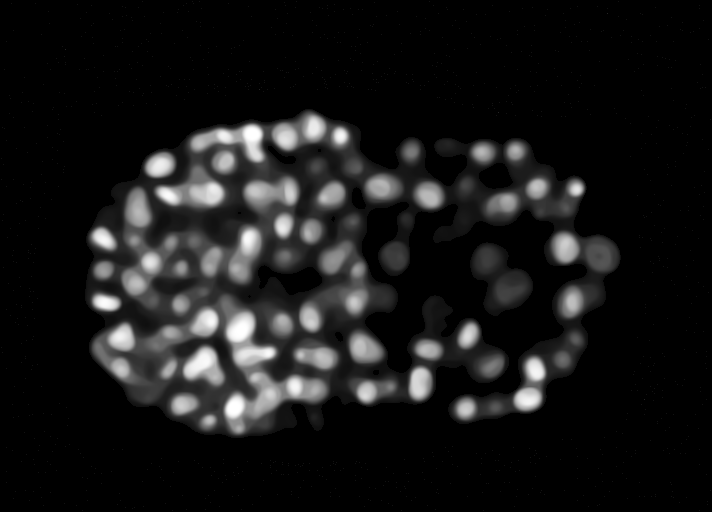

Supplement: Additional file 3 — The program based our method and a sample manual. [file 1471-2105-14-328-S3.zip › Additional_file3/processed_image/130108NHR25p1_L1-t193-p24.tif]

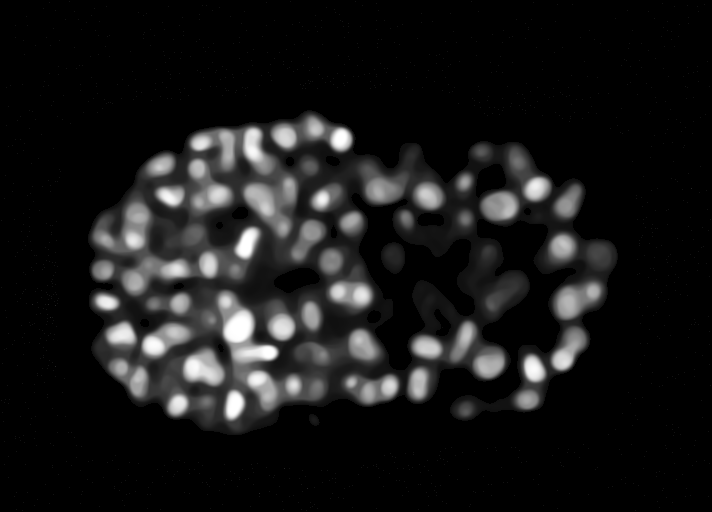

Supplement: Additional file 3 — The program based our method and a sample manual. [file 1471-2105-14-328-S3.zip › Additional_file3/processed_image/130108NHR25p1_L1-t193-p25.tif]

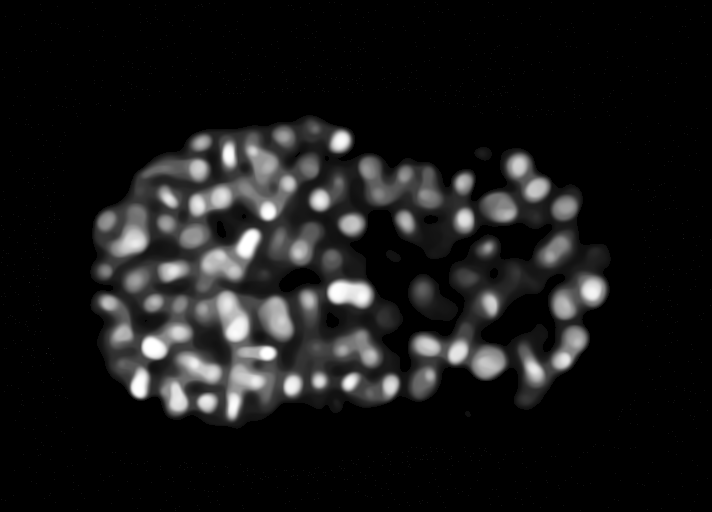

Supplement: Additional file 3 — The program based our method and a sample manual. [file 1471-2105-14-328-S3.zip › Additional_file3/processed_image/130108NHR25p1_L1-t193-p26.tif]

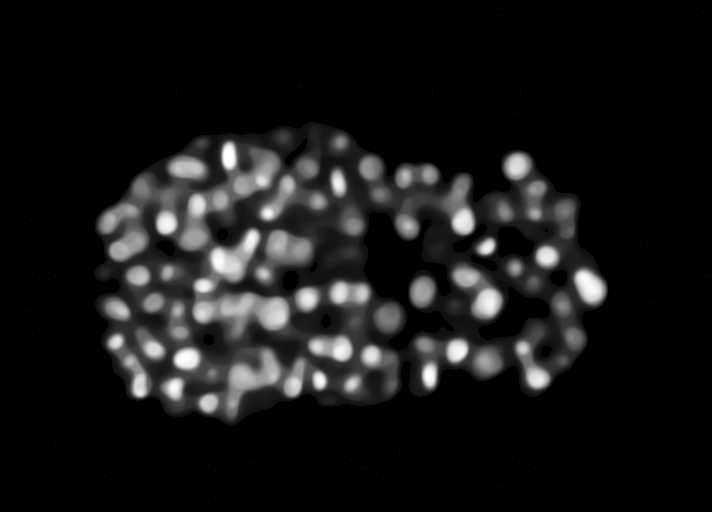

Supplement: Additional file 3 — The program based our method and a sample manual. [file 1471-2105-14-328-S3.zip › Additional_file3/processed_image/130108NHR25p1_L1-t193-p27.tif]

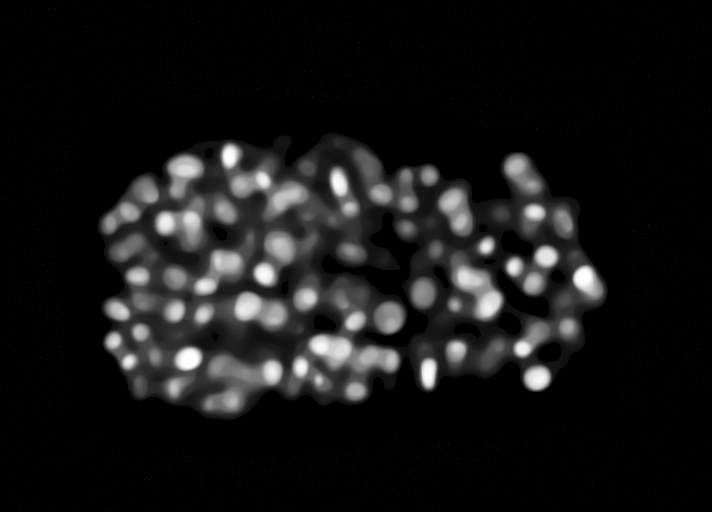

Supplement: Additional file 3 — The program based our method and a sample manual. [file 1471-2105-14-328-S3.zip › Additional_file3/processed_image/130108NHR25p1_L1-t193-p28.tif]

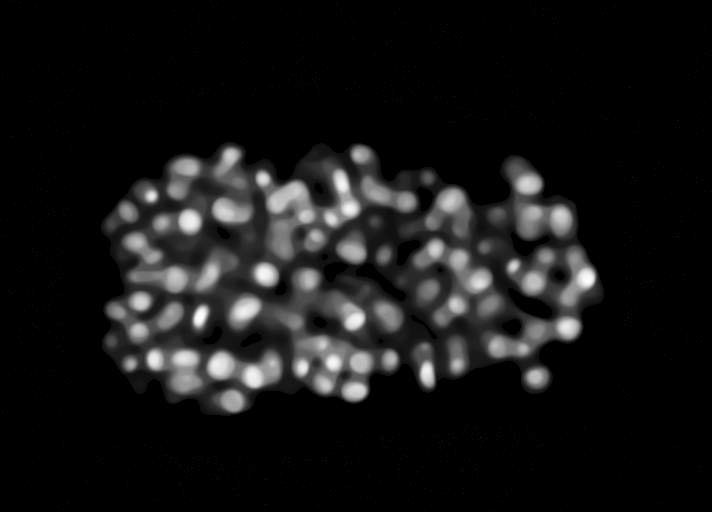

Supplement: Additional file 3 — The program based our method and a sample manual. [file 1471-2105-14-328-S3.zip › Additional_file3/processed_image/130108NHR25p1_L1-t193-p29.tif]

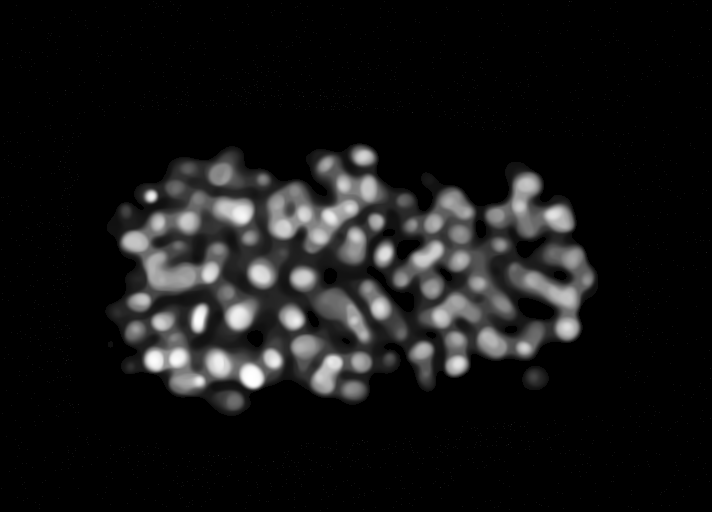

Supplement: Additional file 3 — The program based our method and a sample manual. [file 1471-2105-14-328-S3.zip › Additional_file3/processed_image/130108NHR25p1_L1-t193-p30.tif]

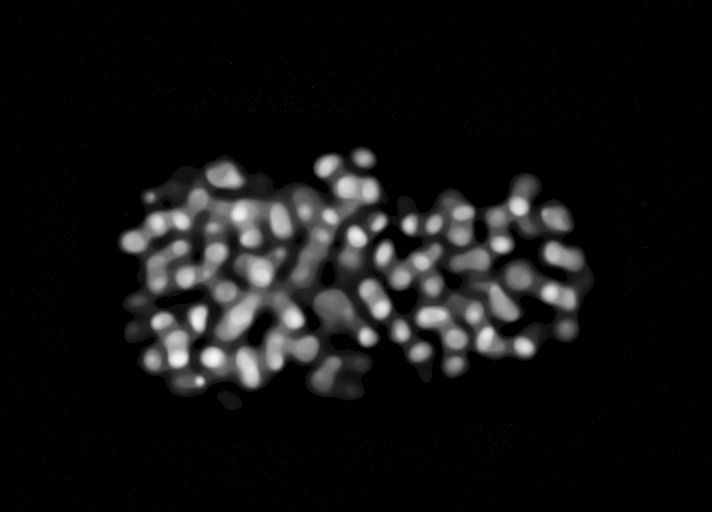

Supplement: Additional file 3 — The program based our method and a sample manual. [file 1471-2105-14-328-S3.zip › Additional_file3/processed_image/130108NHR25p1_L1-t193-p31.tif]

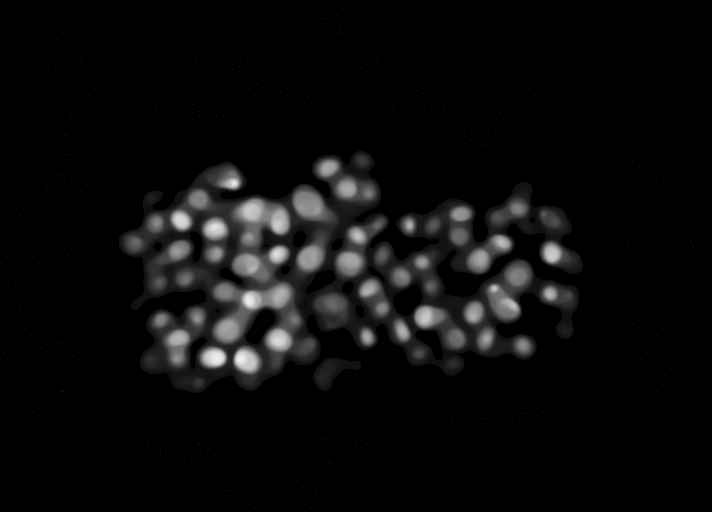

Supplement: Additional file 3 — The program based our method and a sample manual. [file 1471-2105-14-328-S3.zip › Additional_file3/processed_image/130108NHR25p1_L1-t193-p32.tif]

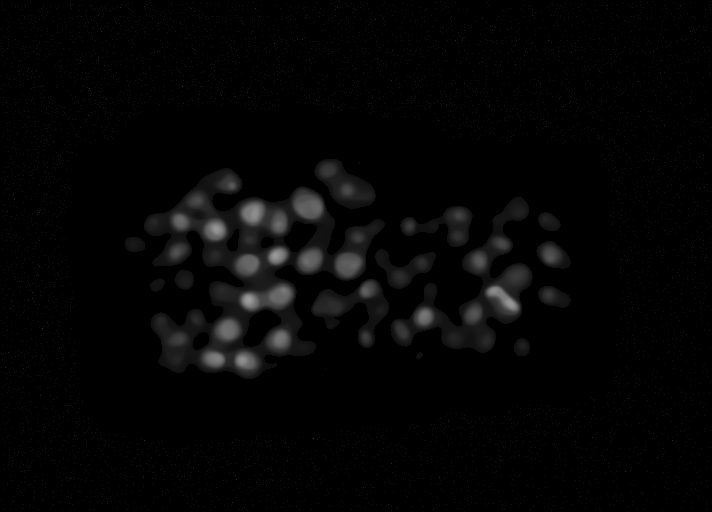

Supplement: Additional file 3 — The program based our method and a sample manual. [file 1471-2105-14-328-S3.zip › Additional_file3/processed_image/130108NHR25p1_L1-t193-p33.tif]

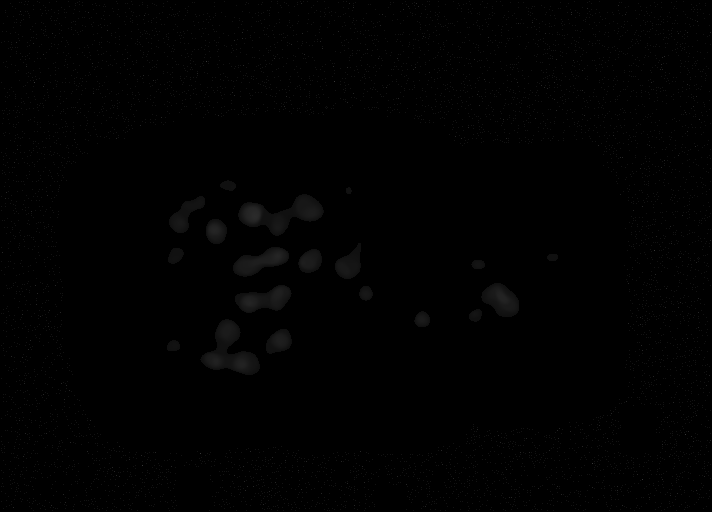

Supplement: Additional file 3 — The program based our method and a sample manual. [file 1471-2105-14-328-S3.zip › Additional_file3/processed_image/130108NHR25p1_L1-t193-p34.tif]

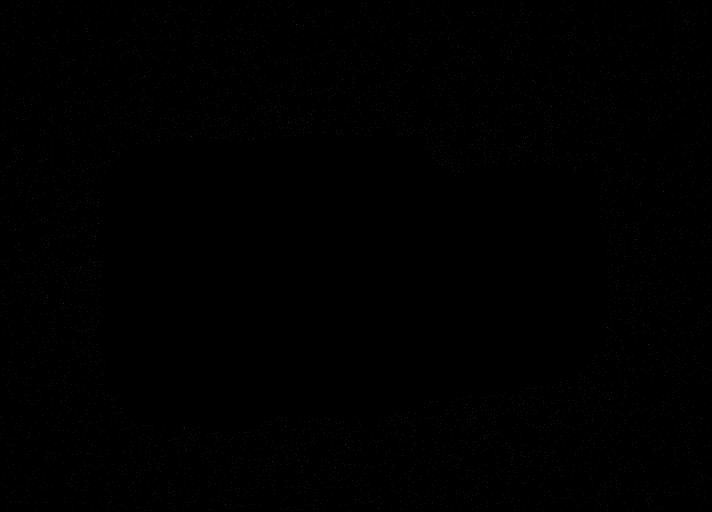

Supplement: Additional file 3 — The program based our method and a sample manual. [file 1471-2105-14-328-S3.zip › Additional_file3/processed_image/130108NHR25p1_L1-t193-p35.tif]

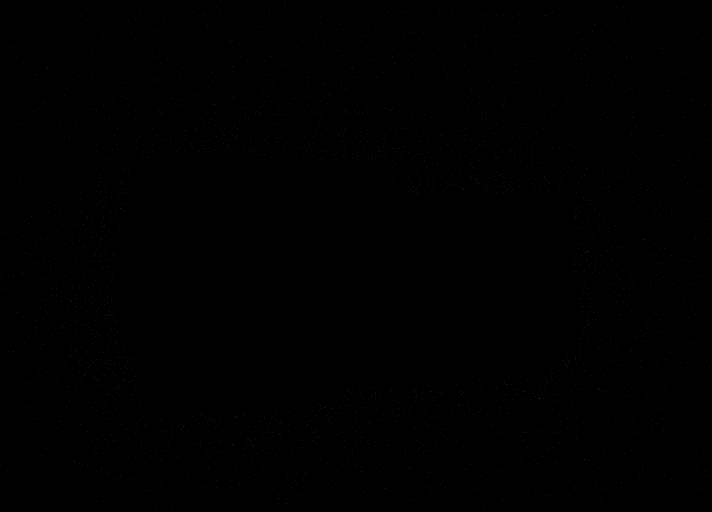

Supplement: Additional file 3 — The program based our method and a sample manual. [file 1471-2105-14-328-S3.zip › Additional_file3/processed_image/130108NHR25p1_L1-t193-p36.tif]

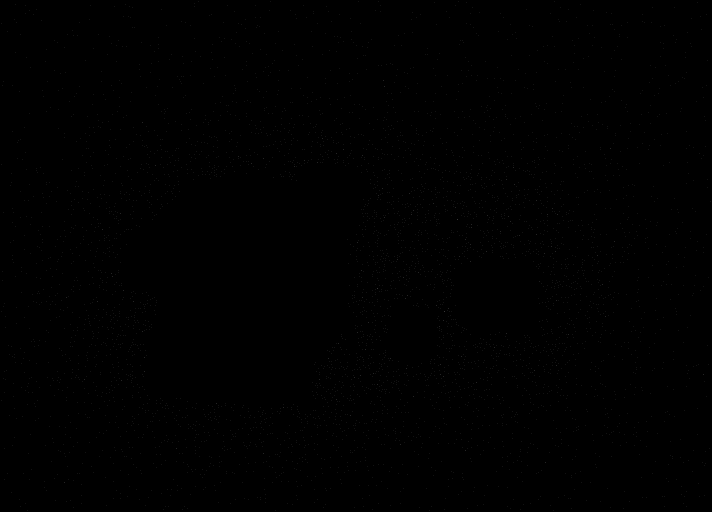

Supplement: Additional file 3 — The program based our method and a sample manual. [file 1471-2105-14-328-S3.zip › Additional_file3/processed_image/130108NHR25p1_L1-t193-p37.tif]

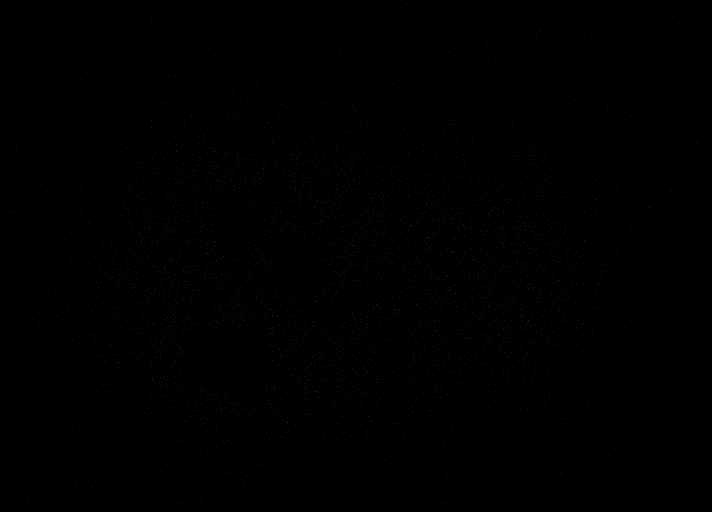

Supplement: Additional file 3 — The program based our method and a sample manual. [file 1471-2105-14-328-S3.zip › Additional_file3/processed_image/130108NHR25p1_L1-t193-p38.tif]

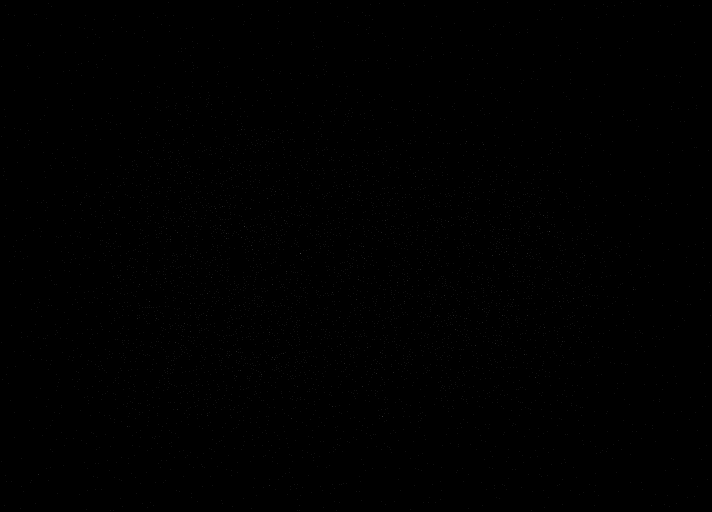

Supplement: Additional file 3 — The program based our method and a sample manual. [file 1471-2105-14-328-S3.zip › Additional_file3/processed_image/130108NHR25p1_L1-t193-p39.tif]

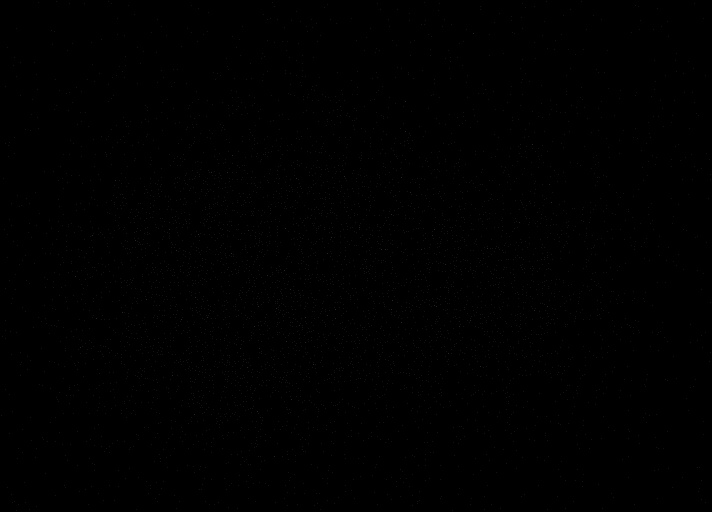

Supplement: Additional file 3 — The program based our method and a sample manual. [file 1471-2105-14-328-S3.zip › Additional_file3/processed_image/130108NHR25p1_L1-t193-p40.tif]

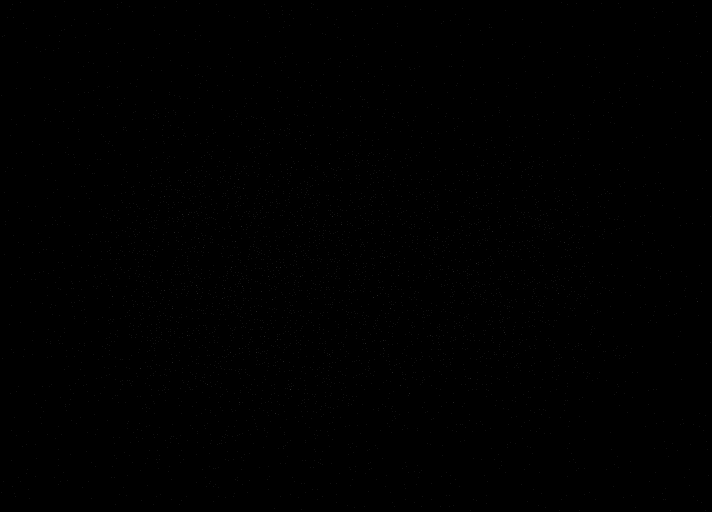

Supplement: Additional file 3 — The program based our method and a sample manual. [file 1471-2105-14-328-S3.zip › Additional_file3/processed_image/130108NHR25p1_L1-t193-p41.tif]

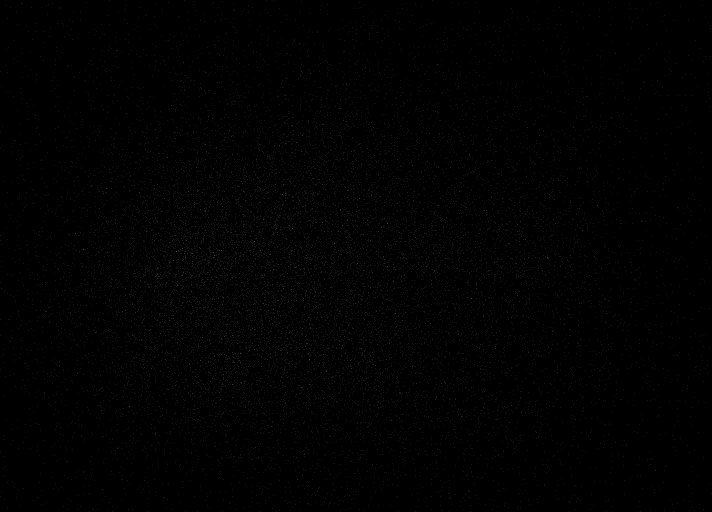

Supplement: Additional file 4 — The program for pre-processing and a sample manual. [file 1471-2105-14-328-S4.zip › Additional_file4_preprocessing_program/original_image/130108NHR25p1_L1-t193-p01.tif]

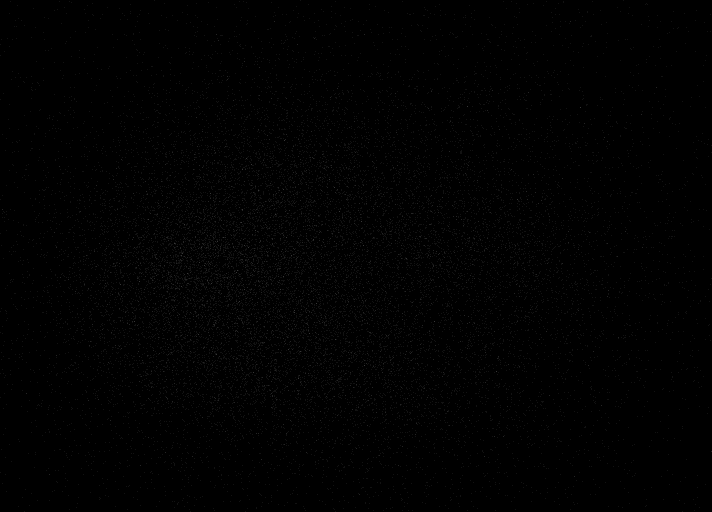

Supplement: Additional file 4 — The program for pre-processing and a sample manual. [file 1471-2105-14-328-S4.zip › Additional_file4_preprocessing_program/original_image/130108NHR25p1_L1-t193-p02.tif]

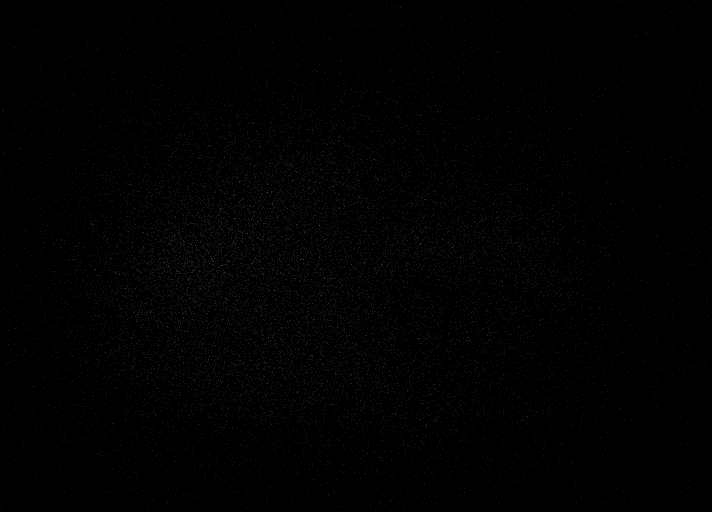

Supplement: Additional file 4 — The program for pre-processing and a sample manual. [file 1471-2105-14-328-S4.zip › Additional_file4_preprocessing_program/original_image/130108NHR25p1_L1-t193-p03.tif]

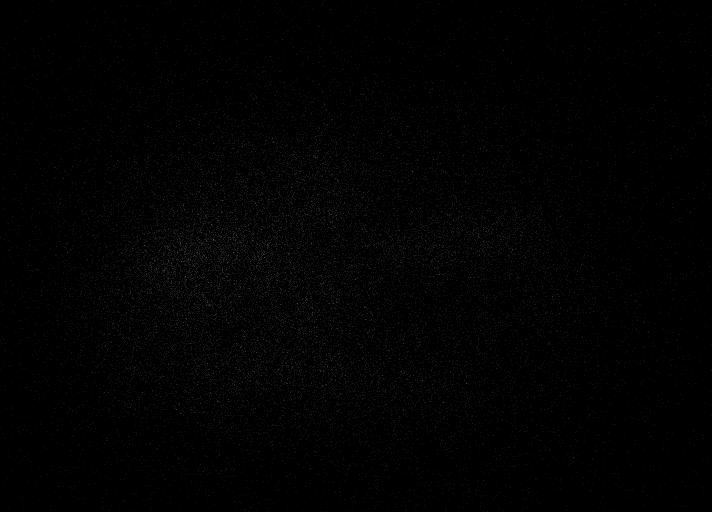

Supplement: Additional file 4 — The program for pre-processing and a sample manual. [file 1471-2105-14-328-S4.zip › Additional_file4_preprocessing_program/original_image/130108NHR25p1_L1-t193-p04.tif]

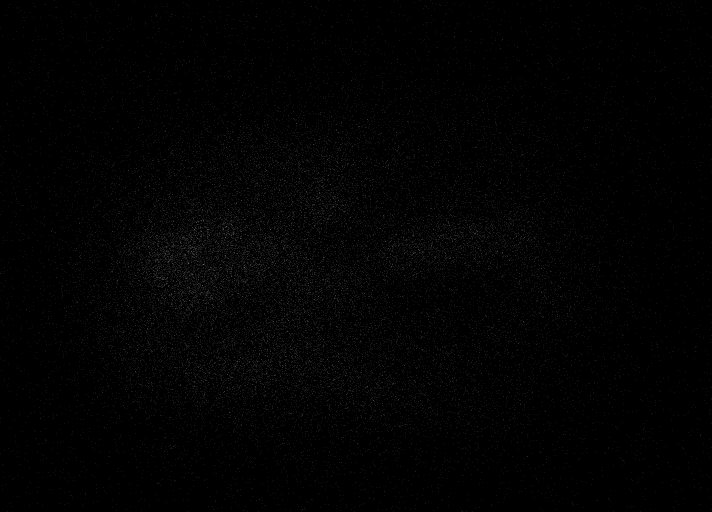

Supplement: Additional file 4 — The program for pre-processing and a sample manual. [file 1471-2105-14-328-S4.zip › Additional_file4_preprocessing_program/original_image/130108NHR25p1_L1-t193-p05.tif]

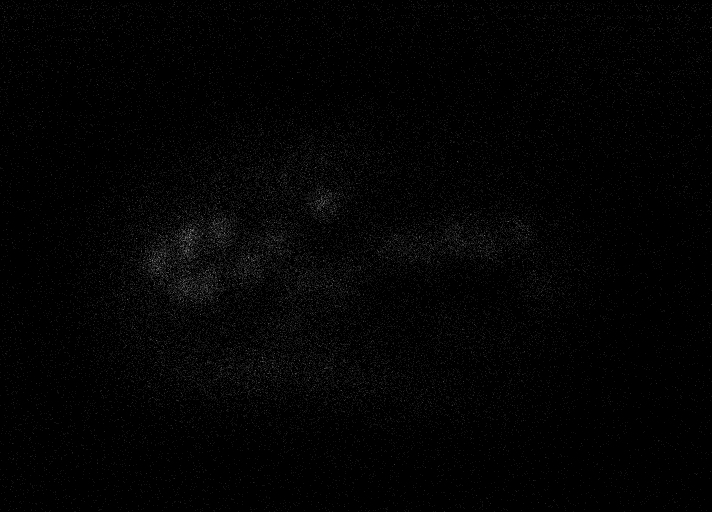

Supplement: Additional file 4 — The program for pre-processing and a sample manual. [file 1471-2105-14-328-S4.zip › Additional_file4_preprocessing_program/original_image/130108NHR25p1_L1-t193-p06.tif]

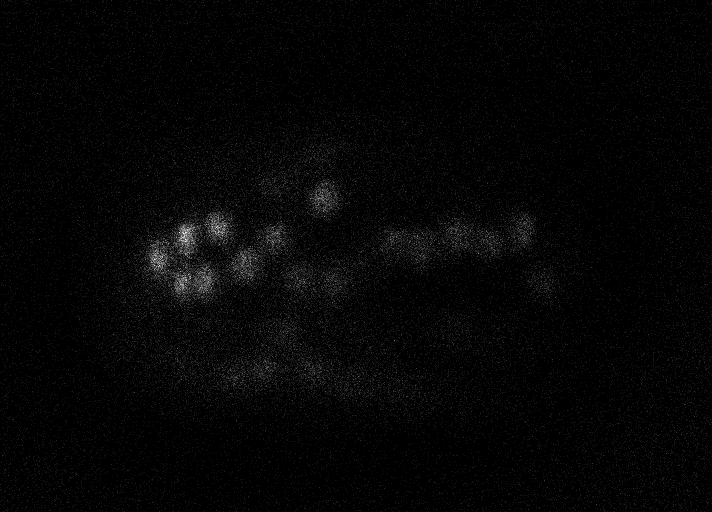

Supplement: Additional file 4 — The program for pre-processing and a sample manual. [file 1471-2105-14-328-S4.zip › Additional_file4_preprocessing_program/original_image/130108NHR25p1_L1-t193-p07.tif]

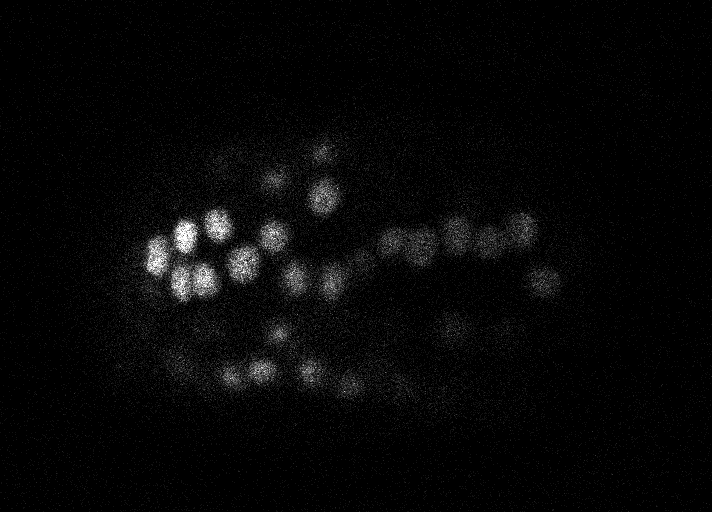

Supplement: Additional file 4 — The program for pre-processing and a sample manual. [file 1471-2105-14-328-S4.zip › Additional_file4_preprocessing_program/original_image/130108NHR25p1_L1-t193-p08.tif]

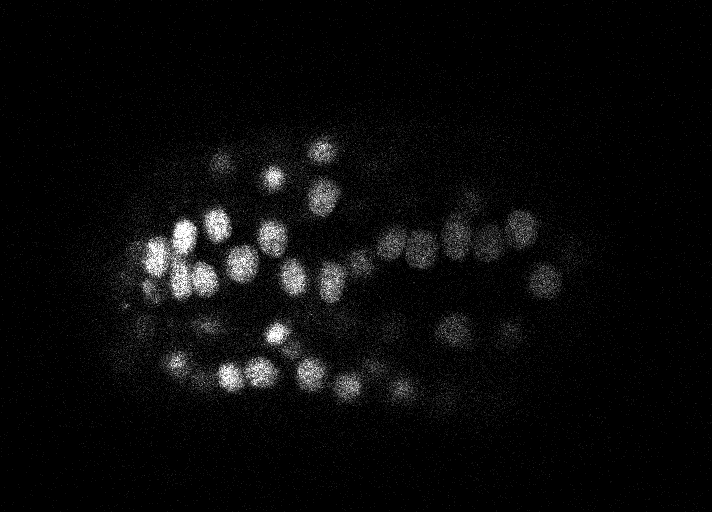

Supplement: Additional file 4 — The program for pre-processing and a sample manual. [file 1471-2105-14-328-S4.zip › Additional_file4_preprocessing_program/original_image/130108NHR25p1_L1-t193-p09.tif]

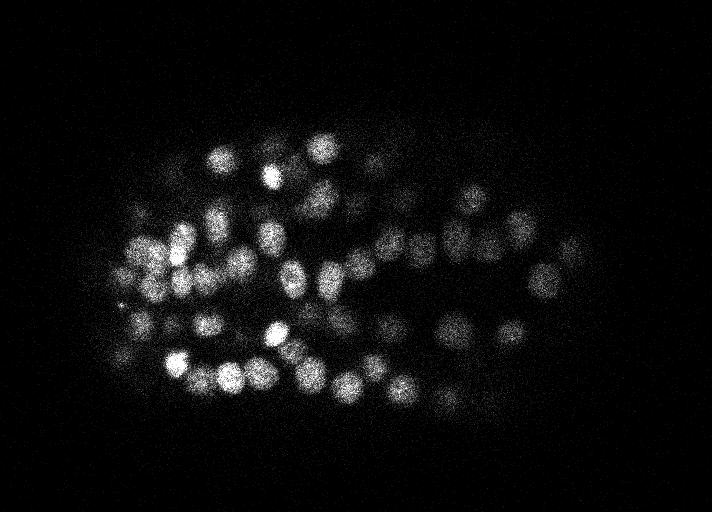

Supplement: Additional file 4 — The program for pre-processing and a sample manual. [file 1471-2105-14-328-S4.zip › Additional_file4_preprocessing_program/original_image/130108NHR25p1_L1-t193-p10.tif]

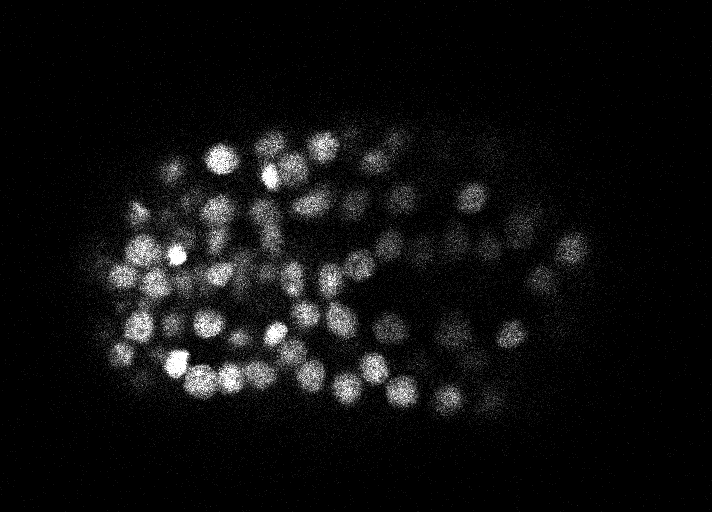

Supplement: Additional file 4 — The program for pre-processing and a sample manual. [file 1471-2105-14-328-S4.zip › Additional_file4_preprocessing_program/original_image/130108NHR25p1_L1-t193-p11.tif]

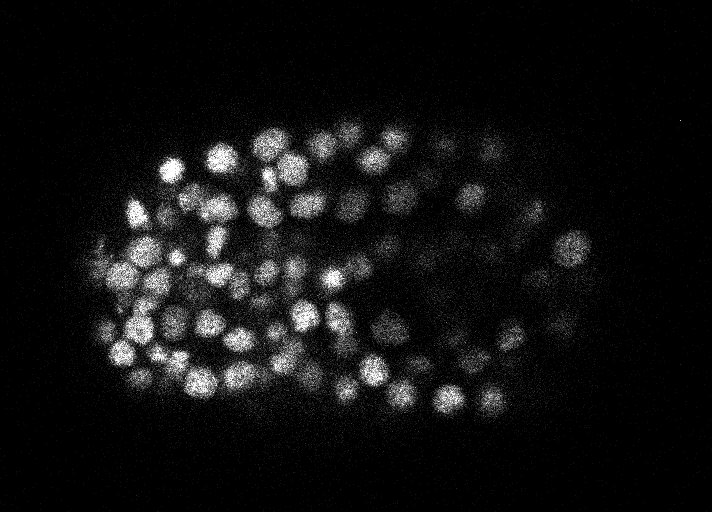

Supplement: Additional file 4 — The program for pre-processing and a sample manual. [file 1471-2105-14-328-S4.zip › Additional_file4_preprocessing_program/original_image/130108NHR25p1_L1-t193-p12.tif]

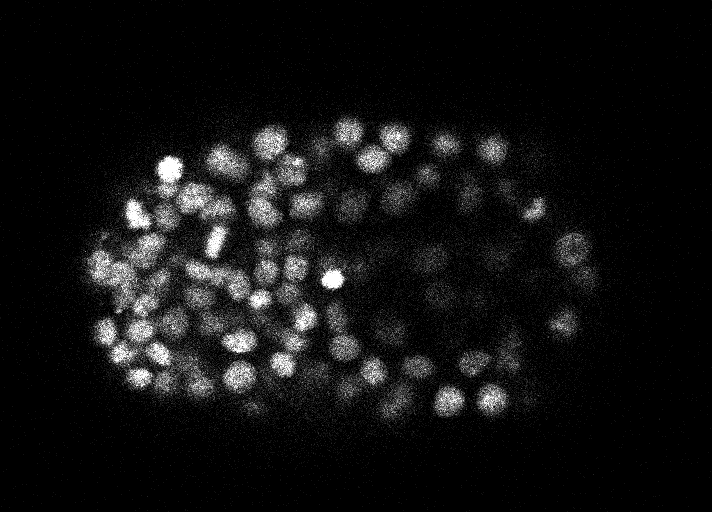

Supplement: Additional file 4 — The program for pre-processing and a sample manual. [file 1471-2105-14-328-S4.zip › Additional_file4_preprocessing_program/original_image/130108NHR25p1_L1-t193-p13.tif]

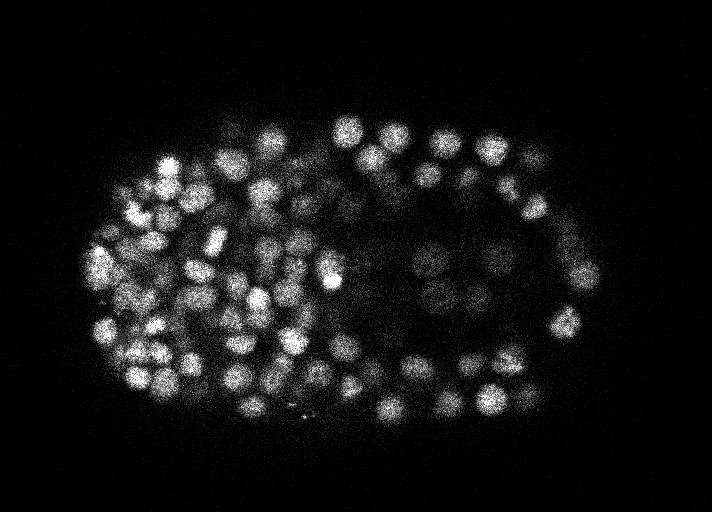

Supplement: Additional file 4 — The program for pre-processing and a sample manual. [file 1471-2105-14-328-S4.zip › Additional_file4_preprocessing_program/original_image/130108NHR25p1_L1-t193-p14.tif]

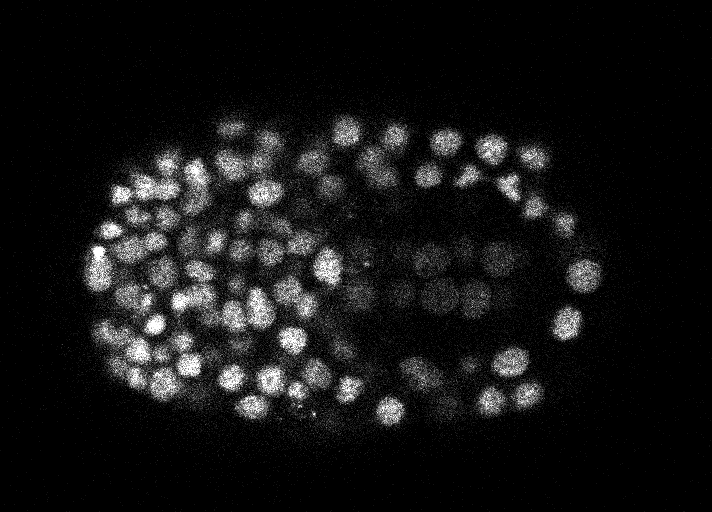

Supplement: Additional file 4 — The program for pre-processing and a sample manual. [file 1471-2105-14-328-S4.zip › Additional_file4_preprocessing_program/original_image/130108NHR25p1_L1-t193-p15.tif]

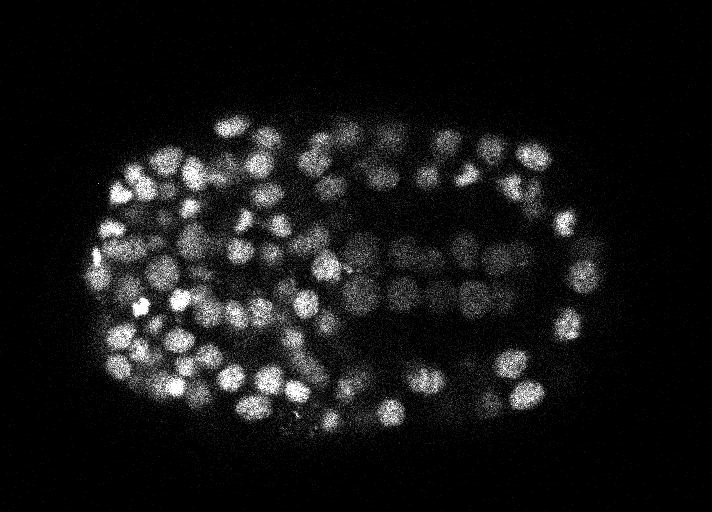

Supplement: Additional file 4 — The program for pre-processing and a sample manual. [file 1471-2105-14-328-S4.zip › Additional_file4_preprocessing_program/original_image/130108NHR25p1_L1-t193-p16.tif]

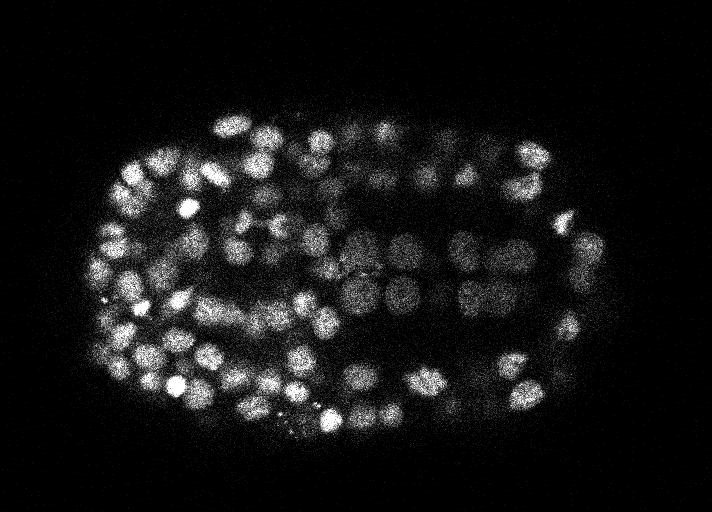

Supplement: Additional file 4 — The program for pre-processing and a sample manual. [file 1471-2105-14-328-S4.zip › Additional_file4_preprocessing_program/original_image/130108NHR25p1_L1-t193-p17.tif]

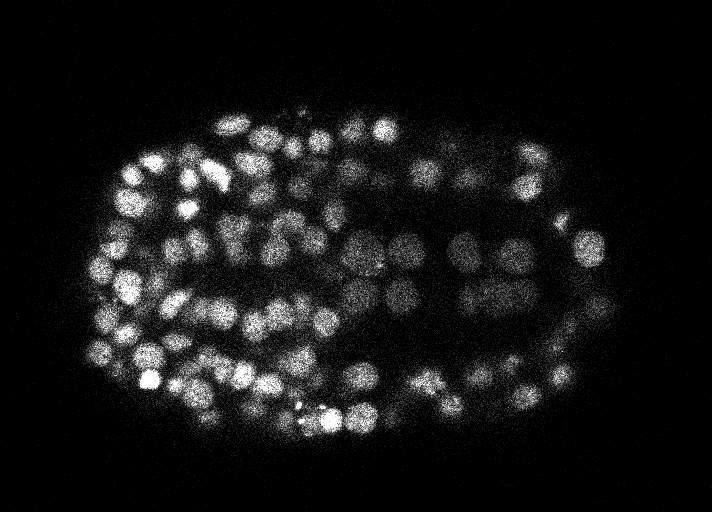

Supplement: Additional file 4 — The program for pre-processing and a sample manual. [file 1471-2105-14-328-S4.zip › Additional_file4_preprocessing_program/original_image/130108NHR25p1_L1-t193-p18.tif]

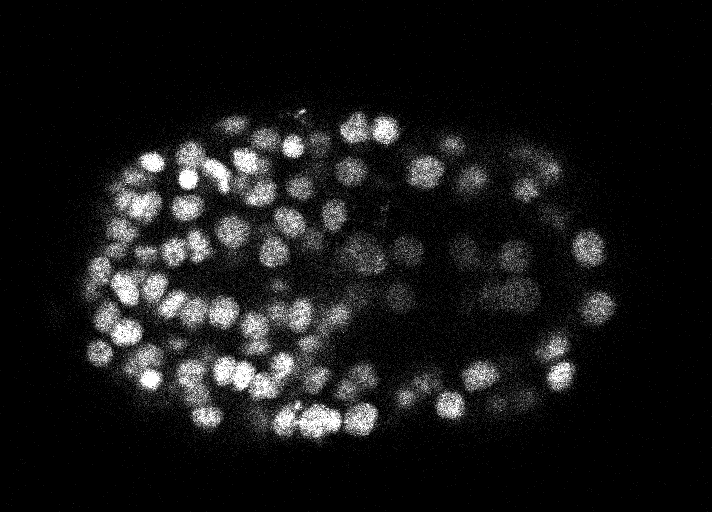

Supplement: Additional file 4 — The program for pre-processing and a sample manual. [file 1471-2105-14-328-S4.zip › Additional_file4_preprocessing_program/original_image/130108NHR25p1_L1-t193-p19.tif]

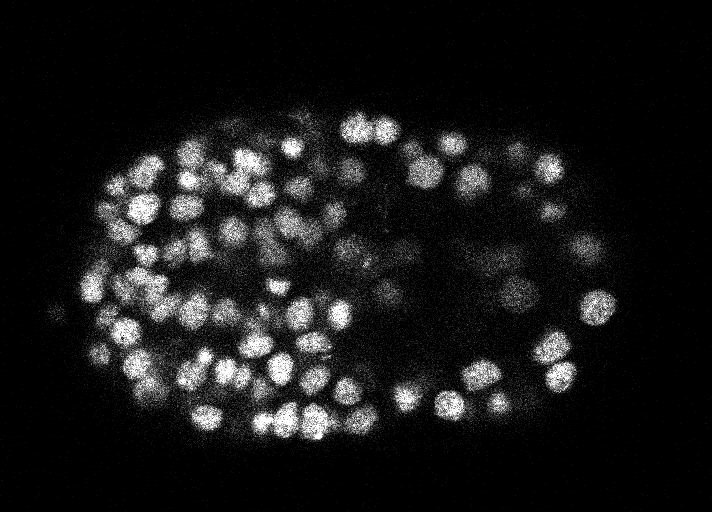

Supplement: Additional file 4 — The program for pre-processing and a sample manual. [file 1471-2105-14-328-S4.zip › Additional_file4_preprocessing_program/original_image/130108NHR25p1_L1-t193-p20.tif]

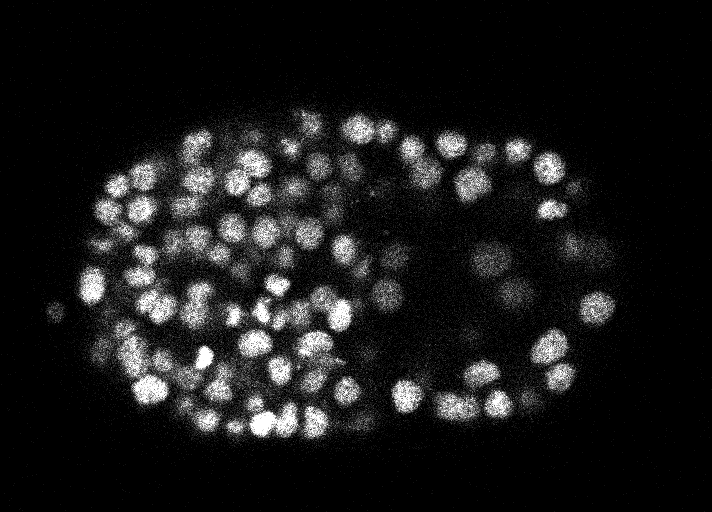

Supplement: Additional file 4 — The program for pre-processing and a sample manual. [file 1471-2105-14-328-S4.zip › Additional_file4_preprocessing_program/original_image/130108NHR25p1_L1-t193-p21.tif]

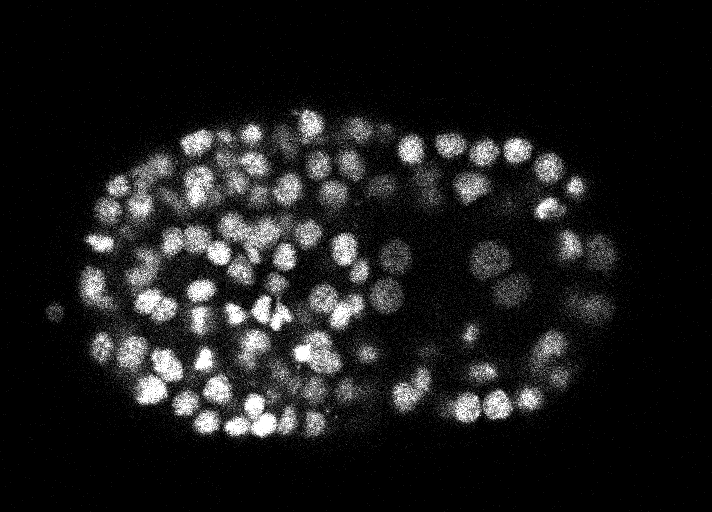

Supplement: Additional file 4 — The program for pre-processing and a sample manual. [file 1471-2105-14-328-S4.zip › Additional_file4_preprocessing_program/original_image/130108NHR25p1_L1-t193-p22.tif]

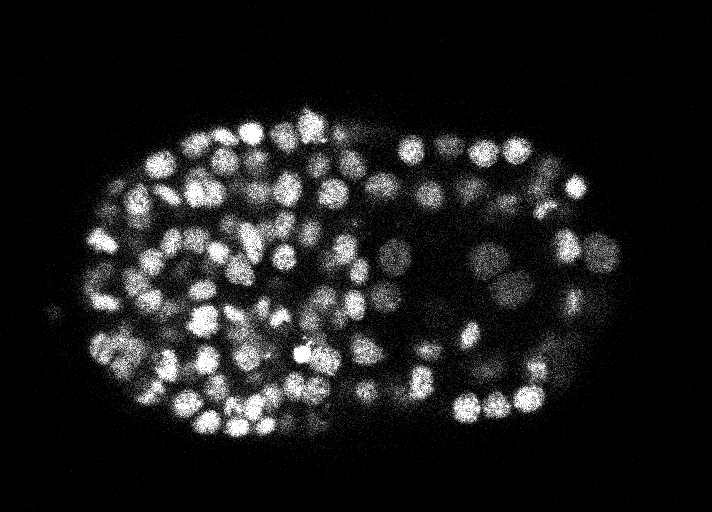

Supplement: Additional file 4 — The program for pre-processing and a sample manual. [file 1471-2105-14-328-S4.zip › Additional_file4_preprocessing_program/original_image/130108NHR25p1_L1-t193-p23.tif]

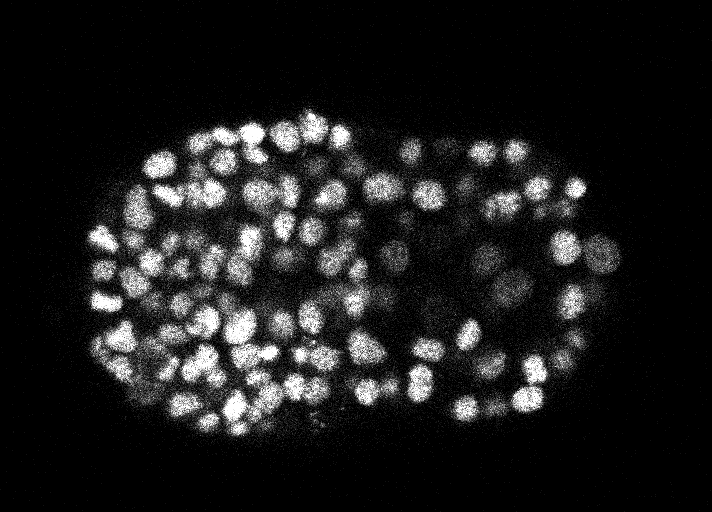

Supplement: Additional file 4 — The program for pre-processing and a sample manual. [file 1471-2105-14-328-S4.zip › Additional_file4_preprocessing_program/original_image/130108NHR25p1_L1-t193-p24.tif]

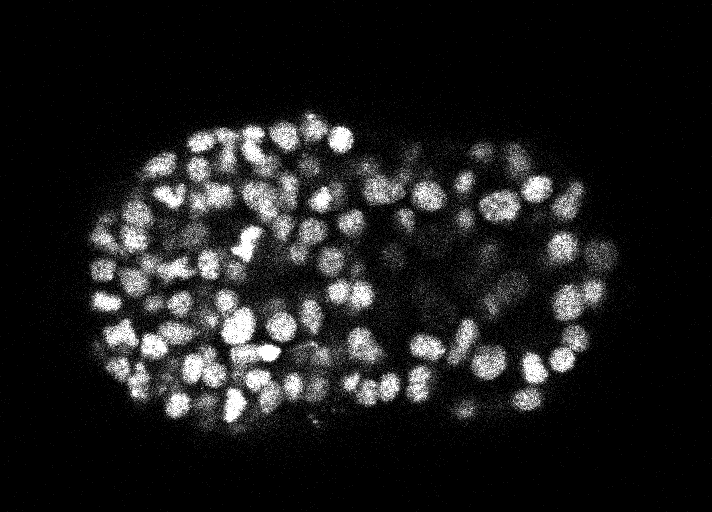

Supplement: Additional file 4 — The program for pre-processing and a sample manual. [file 1471-2105-14-328-S4.zip › Additional_file4_preprocessing_program/original_image/130108NHR25p1_L1-t193-p25.tif]

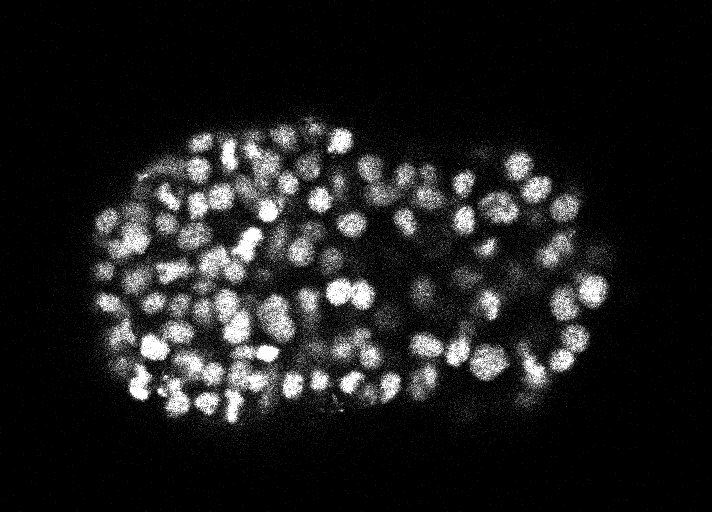

Supplement: Additional file 4 — The program for pre-processing and a sample manual. [file 1471-2105-14-328-S4.zip › Additional_file4_preprocessing_program/original_image/130108NHR25p1_L1-t193-p26.tif]

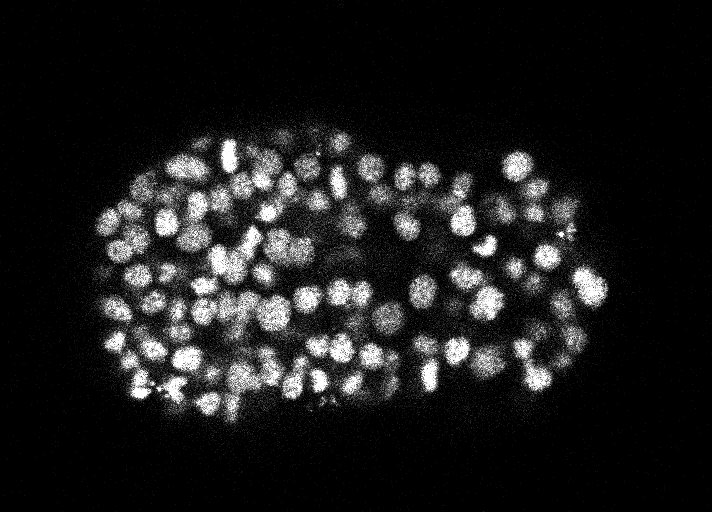

Supplement: Additional file 4 — The program for pre-processing and a sample manual. [file 1471-2105-14-328-S4.zip › Additional_file4_preprocessing_program/original_image/130108NHR25p1_L1-t193-p27.tif]

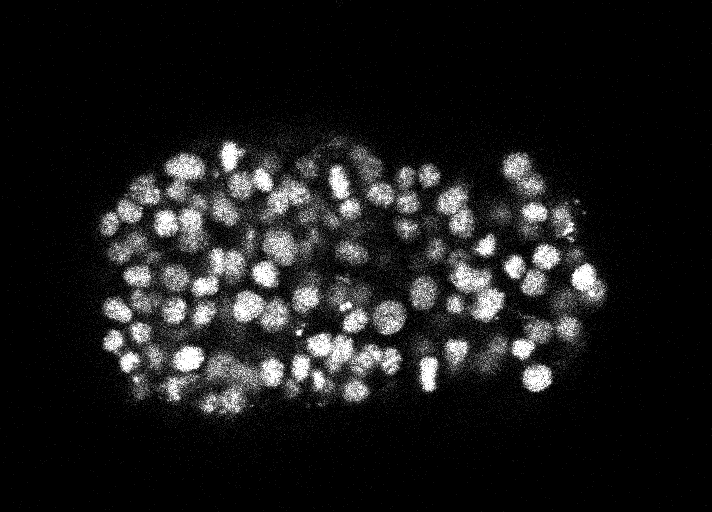

Supplement: Additional file 4 — The program for pre-processing and a sample manual. [file 1471-2105-14-328-S4.zip › Additional_file4_preprocessing_program/original_image/130108NHR25p1_L1-t193-p28.tif]

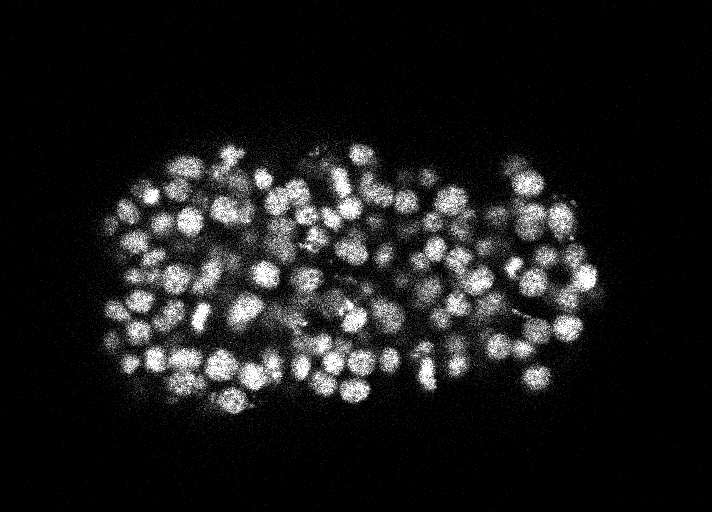

Supplement: Additional file 4 — The program for pre-processing and a sample manual. [file 1471-2105-14-328-S4.zip › Additional_file4_preprocessing_program/original_image/130108NHR25p1_L1-t193-p29.tif]

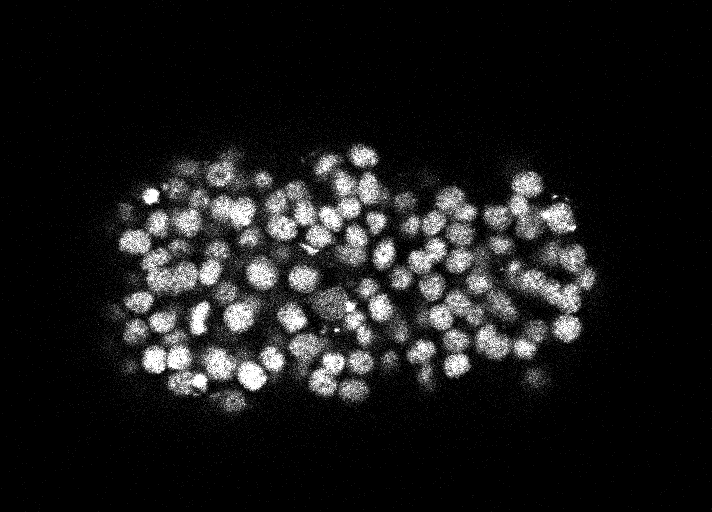

Supplement: Additional file 4 — The program for pre-processing and a sample manual. [file 1471-2105-14-328-S4.zip › Additional_file4_preprocessing_program/original_image/130108NHR25p1_L1-t193-p30.tif]

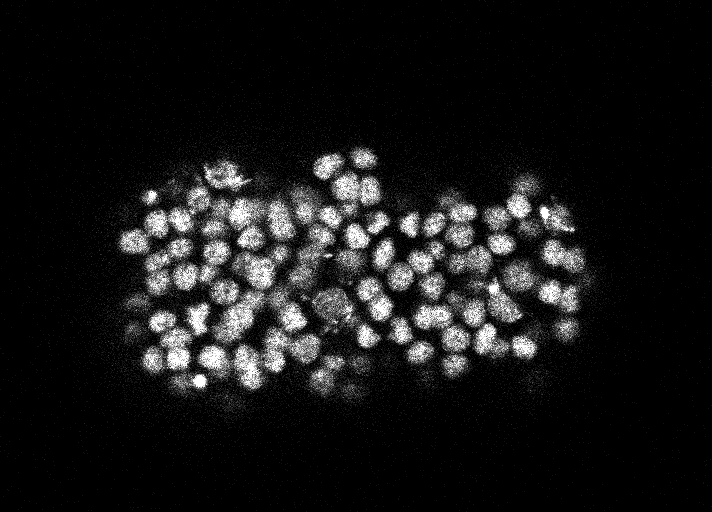

Supplement: Additional file 4 — The program for pre-processing and a sample manual. [file 1471-2105-14-328-S4.zip › Additional_file4_preprocessing_program/original_image/130108NHR25p1_L1-t193-p31.tif]

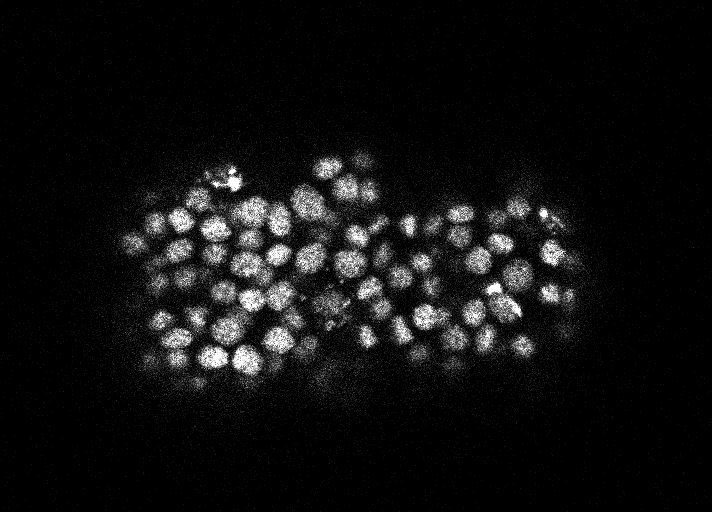

Supplement: Additional file 4 — The program for pre-processing and a sample manual. [file 1471-2105-14-328-S4.zip › Additional_file4_preprocessing_program/original_image/130108NHR25p1_L1-t193-p32.tif]

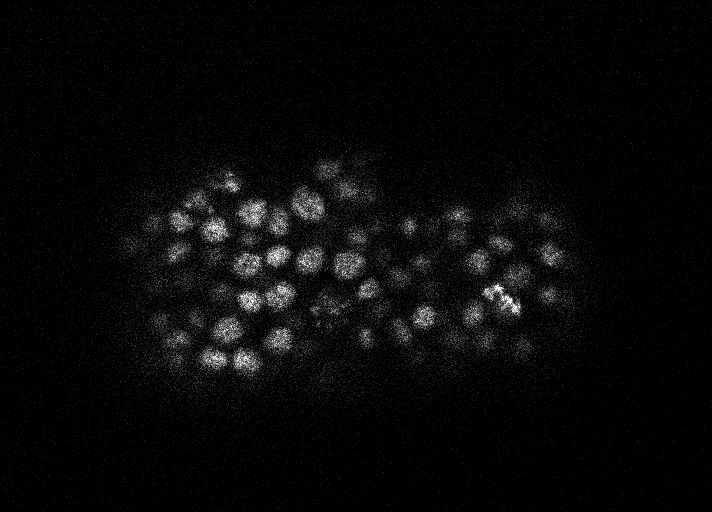

Supplement: Additional file 4 — The program for pre-processing and a sample manual. [file 1471-2105-14-328-S4.zip › Additional_file4_preprocessing_program/original_image/130108NHR25p1_L1-t193-p33.tif]

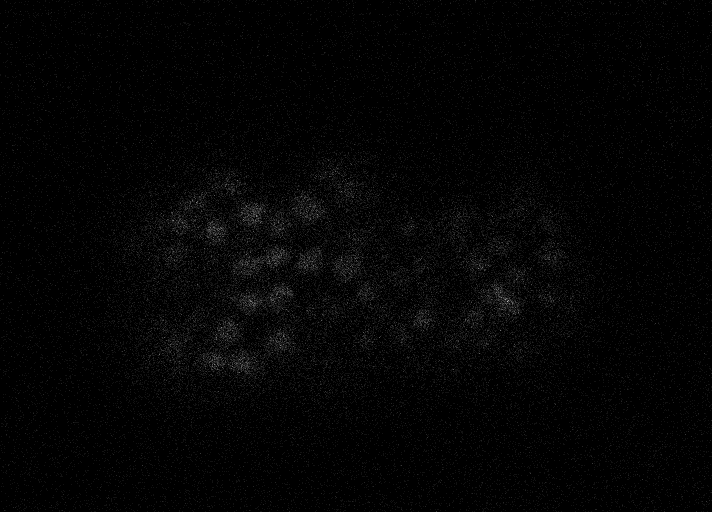

Supplement: Additional file 4 — The program for pre-processing and a sample manual. [file 1471-2105-14-328-S4.zip › Additional_file4_preprocessing_program/original_image/130108NHR25p1_L1-t193-p34.tif]

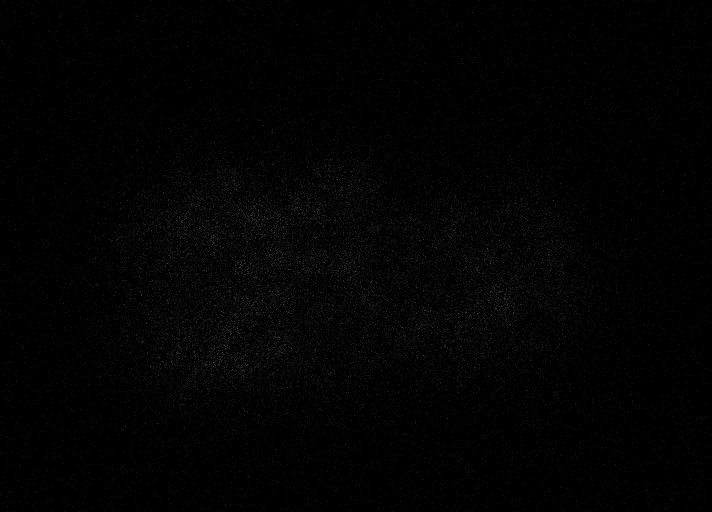

Supplement: Additional file 4 — The program for pre-processing and a sample manual. [file 1471-2105-14-328-S4.zip › Additional_file4_preprocessing_program/original_image/130108NHR25p1_L1-t193-p35.tif]

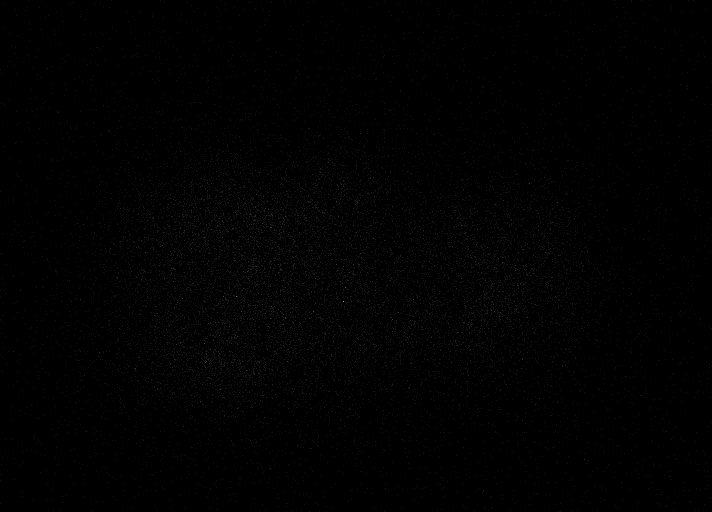

Supplement: Additional file 4 — The program for pre-processing and a sample manual. [file 1471-2105-14-328-S4.zip › Additional_file4_preprocessing_program/original_image/130108NHR25p1_L1-t193-p36.tif]

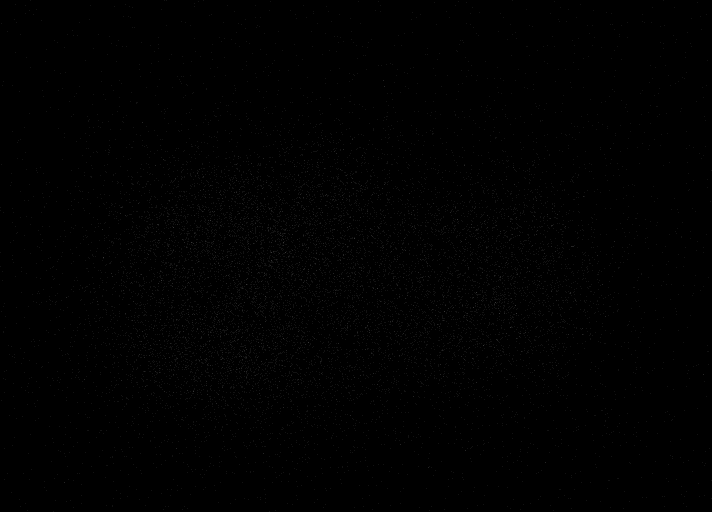

Supplement: Additional file 4 — The program for pre-processing and a sample manual. [file 1471-2105-14-328-S4.zip › Additional_file4_preprocessing_program/original_image/130108NHR25p1_L1-t193-p37.tif]

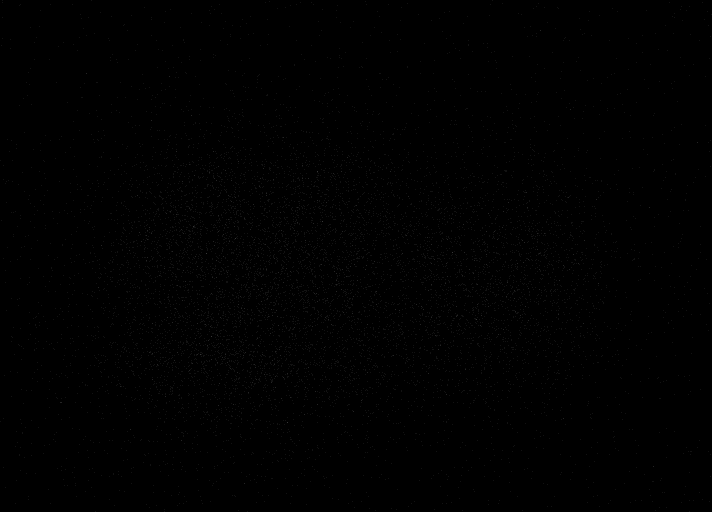

Supplement: Additional file 4 — The program for pre-processing and a sample manual. [file 1471-2105-14-328-S4.zip › Additional_file4_preprocessing_program/original_image/130108NHR25p1_L1-t193-p38.tif]

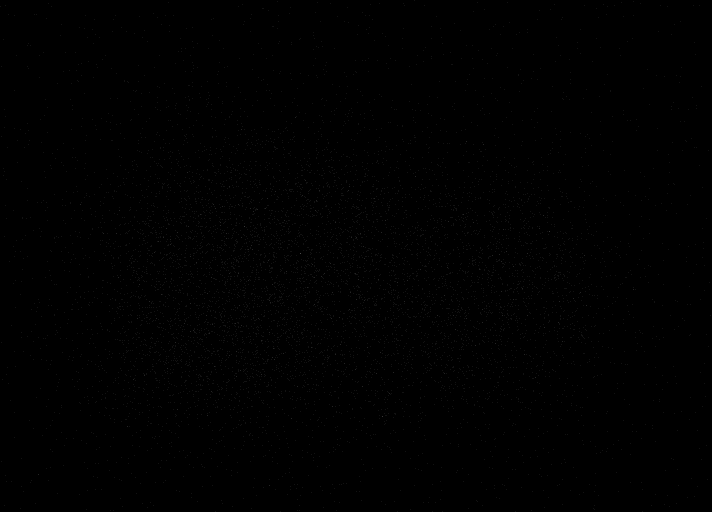

Supplement: Additional file 4 — The program for pre-processing and a sample manual. [file 1471-2105-14-328-S4.zip › Additional_file4_preprocessing_program/original_image/130108NHR25p1_L1-t193-p39.tif]

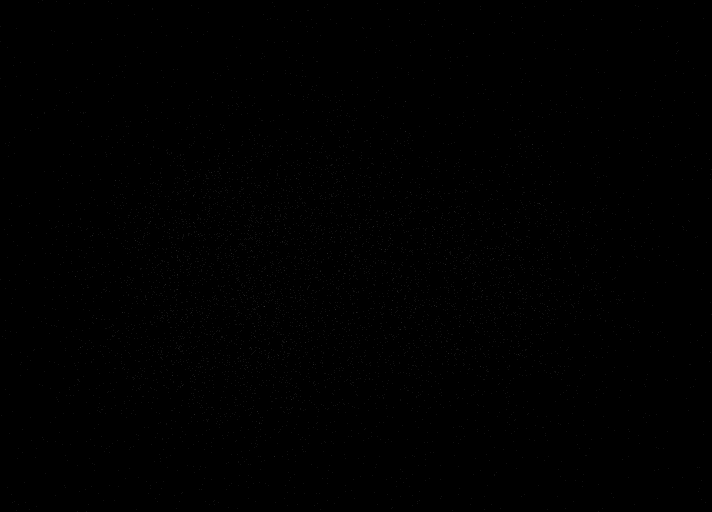

Supplement: Additional file 4 — The program for pre-processing and a sample manual. [file 1471-2105-14-328-S4.zip › Additional_file4_preprocessing_program/original_image/130108NHR25p1_L1-t193-p40.tif]

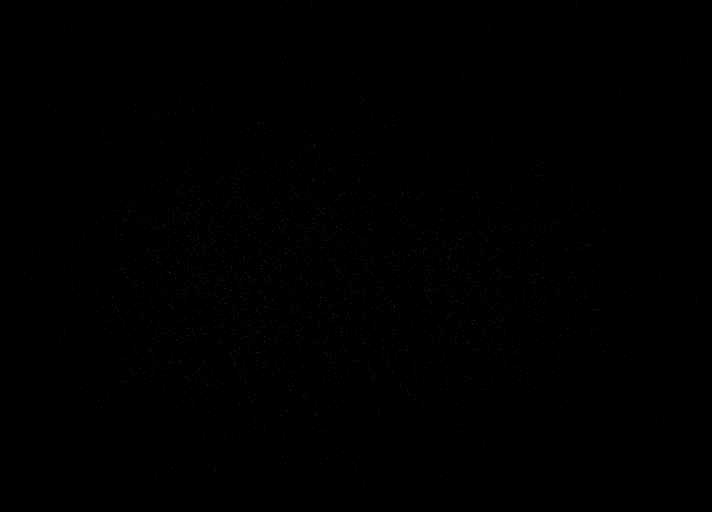

Supplement: Additional file 4 — The program for pre-processing and a sample manual. [file 1471-2105-14-328-S4.zip › Additional_file4_preprocessing_program/original_image/130108NHR25p1_L1-t193-p41.tif]
